# Full-Length Transcriptome of *Myotis pilosus* as a Reference Resource and Mining of Auditory and Immune Related Genes

**DOI:** 10.3390/ijms24010062

**Published:** 2022-12-21

**Authors:** Xue Wang, Mingyue Bao, Ningning Xu, Ruyi Sun, Wentao Dai, Keping Sun, Hui Wang, Jiang Feng

**Affiliations:** 1 College of Life Science, Jilin Agricultural University, Changchun 130118, China; 2 Jilin Provincial Key Laboratory of Animal Resource Conservation and Utilization, Northeast Normal University, Changchun 130117, China

**Keywords:** *Myotis pilosus*, PacBio, full-length transcriptome, SMRT sequencing, isoform

## Abstract

Rickett’s big-footed bat, *Myotis pilosus*, which belongs to the family Vespertilionida, is the only known piscivorous bat in East Asia. Accurate whole genome and transcriptome annotations are essential for the study of bat biological evolution. The lack of a whole genome for *M. pilosus* has limited our understanding of the molecular mechanisms underlying the species’ evolution, echolocation, and immune response. In the present work, we sequenced the entire transcriptome using error-corrected PacBio single-molecule real-time (SMRT) data. Then, a total of 40 GB of subreads were generated, including 29,991 full-length non-chimeric (FLNC) sequences. After correction by Illumina short reads and de-redundancy, we obtained 26,717 error-corrected isoforms with an average length of 3018.91 bp and an N50 length of 3447 bp. A total of 1528 alternative splicing (AS) events were detected by transcriptome structural analysis. Furthermore, 1032 putative transcription factors (TFs) were identified, with additional identification of several long non-coding RNAs (lncRNAs) with high confidence. Moreover, several key genes, including *PRL-2*, *DPP4*, *Glul*, and *ND1* were also identified as being associated with metabolism, immunity, nervous system processes, and auditory perception. A multitude of pattern recognition receptors was identified, including NLR, RLR, SRCR, the antiviral molecule IRF3, and the IFN receptor subunit IFNAR1. High-quality reference genomes at the transcriptome level may be used to quantify gene or transcript expression, evaluate alternative splicing levels, identify novel transcripts, and enhance genome annotation in bats.

## 1. Introduction

With more than 1440 species of bats described worldwide, the order Chiroptera is second only to rodents in ecological and taxonomic diversity. Bats occupy a vast niche in the night sky, their diet is complex and diverse, and *Myotis pilosus*, a fish-eating bat, is the most striking member of the group. The East Asian fish-eating bat is large-footed and has well-developed claws, *M. pilosus*, uses a 37.78 ± 1.04 kHz downward frequency-modulated (FM) sweep for echolocation. This fish-eating bat can utilize echolocation to accurately detect ripples produced by fish on the surface of the water, making it an excellent model for studying high-frequency hearing and the coevolution of bats and fish [1]. In addition, increasing numbers of studies have reported that bats are potential hosts for viruses, and thus, their unique immune systems and high immune tolerance have received extensive attention [2]. However, there is still a lack of adequate genomic data for *M. pilosus*, and this limits the in-depth research into the ecological adaptation of fish-eating bats represented by *M. pilosus*. Therefore, it is crucial to perform further research on the *M. pilosus* genome and transcriptome.

In recent years, numerous studies from multiple omics levels have been conducted on niche expansion of feeding habits, immunologically privileged sites for microbial community residence in bats, brain metabolism during hibernation, and hearing genes in echolocation, revealing the genetic basis and ecological adaptation mechanism of *M. pilosus* from various perspectives. For example, Chang et al. [3] investigated the dietary composition and seasonality of *M. pilosus* and *M. fimbriatus* using DNA meta-barcoding. Their findings showed that variation in insect abundance or interspecific competition may not have had a significant impact on *M. pilosus*’ dietary expansion from insects to fish. Fang et al. [4] identified and described an immunosuppressant protein (MTX) that is highly concentrated in the submandibular salivary gland of *M. pilosus*. The presence of high levels of MTX in the salivary glands of bats suggests a mechanism for the development of immunological privilege and immune tolerance, as well as evidence of viral shedding via oral secretions. Zhang [5] compared the brain proteins of active and torpid *M. pilosus* bats by a proteomic approach and found that torpid bats used energy produced from anaerobic glycolysis and PTMs to adjust enzyme activities critical for TCA cycle efflux. In addition, some work has been carried out at the transcriptome level. Dong et al. [6] compared the inner ear expression difference between the *M. pilosus* (an echolocating bat) and *Cynopterus sphinx* (a non-echolocating bat). The results showed that the biological implications of upregulated genes in *M. pilosus* were significantly over-represented in biological process categories, consistent with the inner ear morphological and physiological differentiation between the two bat species. Although de novo transcriptome-based bioinformatic techniques can partially address the issue of no reference genome, no high-quality reference genome has been published for *M. pilosus*, a situation that limits our understanding of the molecular mechanisms such as coevolution, echolocation, and immune response.

Although it is possible to refine the relevant biological analysis by sequencing the genomic data of *M. pilosus*, the traditional genome approach is expensive, resulting in the lack of genomic reference information for many species; this limits the in-depth study of species that are beyond the affordability of general research groups. Fortunately, with the emergence of full-length transcriptome sequencing technology and the development of related analytical methods, these research difficulties can be resolved. The full-length transcriptome provides more benefits than the second-generation transcriptome, particularly for species without a reference genome. Using PacBio Iso-seq to construct a species unigene library, the reference genome at the transcriptome level of the species can be obtained without sequence assembly, thereby providing an excellent genetic information basis for subsequent research. PacBio Iso-seq is also used to detect alternative splicing events without reference genome sequences. In the study of bats’ resistance to viruses and suppression of host inflammation [7], alternative splicing is an essential reason for inhibiting NLRP3-mediated inflammation in bats, providing a basis for the special high innate immune tolerance of bats. These studies emphasize alternative splicing, a crucial transcriptional regulation process that is significant to studies of bats.

Taking advantage of this, full-length transcriptome sequencing has been widely used in the study of many species. The sequencing of the skin tissue of the marine mammal Sperm whale [8] and the sequencing of the head kidney tissue of Atlantic salmon, for example, demonstrate that the broad applicability of the full-length transcriptome and the stable and efficient solution of the follow-up series requires the analysis of genomics [9]. Ma et al. used the approach to perform full-length sequencing of the cochlear tissues of echolocating bats such as *M. pilosus*, providing only the transcriptome map and annotation information [10]. However, no further comparative analyses were performed. In the research mentioned above, a single tissue was utilized for transcriptome sequencing, thus limiting its applicability to specific investigations rather than producing a whole transcriptome map for the species. As a result, we propose that tissue samples from various body areas of different species could be utilized for full-length sequencing and that this will totally produce a reference transcriptome map.

In the present work, we performed full-length transcriptome sequencing and bioinformatic analysis by using the Pacific Bioscience Sequel platform and the Illumina HiSeq platform on the multiple tissues of *M. pilosus*. To generate a high-quality reference genome at the transcriptome level and to combine the advantages of the full-length sequencing data for the identification and analysis of novel transcripts and alternative splicing events, we further identified the molecular basis of alternative splicing events and phenotypes associated with various aspects of ecological adaptation in *M. pilosus*, such as the completion of the identification and characterization of novel transcripts related with echolocation and immunity.

These data not only are of practical reference significance in studies aimed at detecting variable splicing types, but also enrich and complement the genomic resources of bats in studies of adaptive evolution. In addition, the results can facilitate a comparative analysis of bat genomes and help reveal the genetic mechanisms underlying bat evolution. The relevant functional genes found by the study can help us understand the specific immune molecular mechanisms of bats and provide a reference for exploring the molecular targets of diseases.

## 2. Results

### 2.1. PacBio Iso-Seq Sequencing Data Analysis

**Figure 1:**
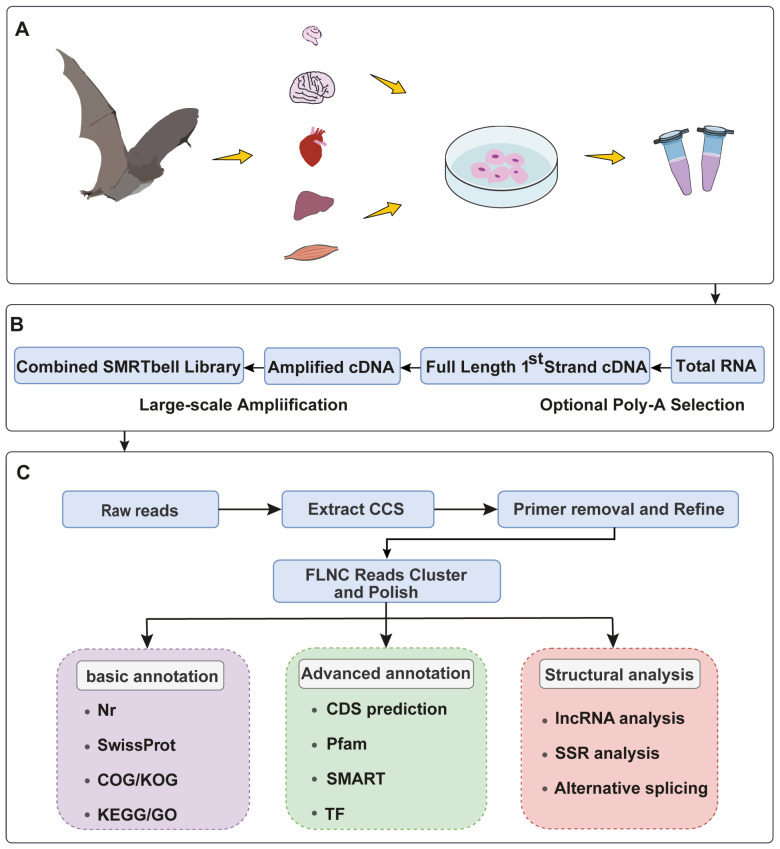
Schematic of the full-length transcriptome reconstruction and analysis pipeline. (**A**) Multiple tissues were extracted from *M. pilosus* and sequenced. (**B**) The workflow of library construction. (**C**) Bioinformatic analysis pipeline.

To reveal the diversity of transcript isoforms, we constructed a full-length RNA-seq library (Figure 1B). Full-length transcripts of *M. pilosus* were characterized according to the analysis process shown in Figure 1. With SMRT sequencing, 40 GB of raw data consisting of 46,086,752,174 total bases were generated. Then, a total of 17,570,601 subreads were obtained, with an average read length of 2622 bp and an N50 of 2926 bp (Table 1, Figure S1A). After self-correction of all subreads, a total of 752,249 CCS were obtained, with an average CCS read length of 2978, (Table 1, Figure S1B). From these, 601,772 full-length non-chimeric reads (FLNC reads) were obtained (Table 1, Figure S1C). The CCS were further corrected to obtain 28,742 high-quality sequences (high-quality isoforms, HQ isoforms, prediction accuracy ≥ 0.99) and 1249 low-quality sequences (low-quality isoforms, LQ isoforms, prediction accuracy < 0.99).

A total of 714 low-quality sequences were corrected after rectification. A total of 29,456 corrected sequences were found with an average read length of 3027.42 bp and an N50 length of 3449 bp. Following correction, redundant sequences were deleted to produce 26,717 isoforms, with an average length of 3018.91 bp and a total length of 80,656,306 bp (Table 1, Figure S1D). The analysis of transcriptome completeness with Benchmarking Universal Single-Copy Orthologs (BUSCO) showed that 39.3% (385) were complete duplicated BUSCOs; 40.3% (395) were complete single-copy BUSCOs; 1.7% (17) were fragmented BUSCO archetypes, and 18.5% (181) were missing BUSCOs (Figure S2). We also discovered that CCS length was longer than in other bats such as *Rhinolophus afnis hainanus* and *Rhinolophus afnis himalayanus* (Figure S3) [10].

### 2.2. Functional Annotation

**Figure 2:**
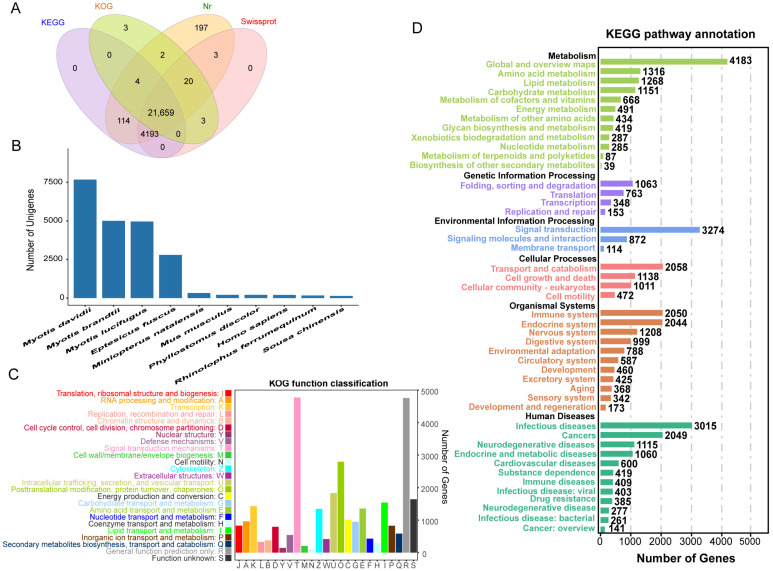
Functional annotation in *M. pilosus*. (**A**) Venn diagram of annotations in NR, KEGG, KOG, and Swiss-Prot databases. (**B**) Distribution of the top 10 species with matched transcripts in the NR database. (**C**) KOG categories of the transcripts. (**D**) KEGG pathways enriched by transcripts.

**Figure 3:**
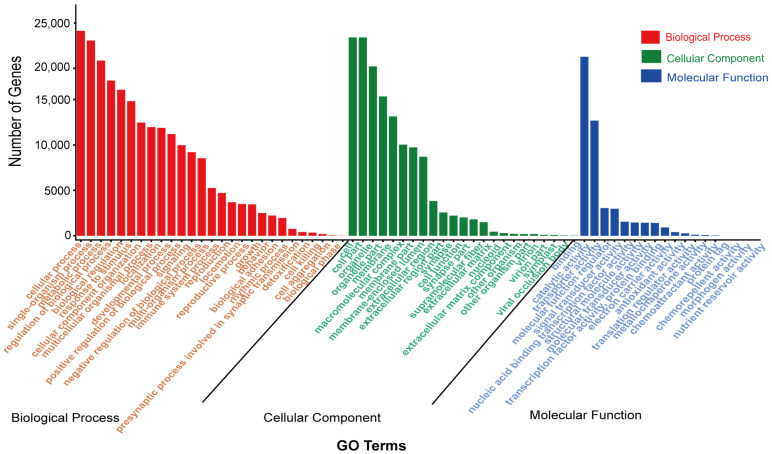
Distribution of GO terms for all annotated transcripts in biological process, cellular component, and molecular function.

All full-length transcripts obtained above were aligned to KEGG, KOG, Nr, and Swiss-Prot databases. A total of 26,198 transcripts were successfully annotated, and for 519 transcripts the annotations were unsuccessful. In total, 39,208 transcripts were annotated in the four databases, of which 26,192, 25,970, 21,691, and 25,878 transcripts were matched to the NR, KEGG, KOG, and Swiss-Prot databases, respectively (Figure 2A). Each transcript was aligned with the homologous sequences of the NR library, to determine which species showed the best sequence comparisons, and we quantified the number of homologous sequences aligned with each species. The top three species with the highest homology were all species of the genus *Myotis*, *Myotis davidii* (7679), *Myotis brandtii* (5007), and *Myotis lucifugus* (4960). *Eptesicus fuscus* (2798), which is a member of the bat family, was also detected (Figure 2B). These species belong to the order Chiroptera, which explains the high similarity of their transcripts to the transcript of *M. pilosus*. The KOG classifications of the transcripts obtained clusters of 25 functional categories (Figure 2C). A total of 4773 transcripts were annotated in signal transduction mechanisms, the most among functional categories. Next was the general function prediction with 4758 transcripts. In KEGG pathways, the transcripts were assigned to six main categories: cellular processes (4706 transcripts), environmental information processing (4260), genetic information processing (2192 transcripts), metabolism (10,237 transcripts), human diseases (10,134 transcripts), and organismal systems (9444 transcripts). Global and overview maps (2129 transcripts) was the largest group of transcripts, followed by signal transduction (1498 transcripts) (Figure 2D). According to GO classification statistics of the transcripts, the annotated results included three broad categories: biological process (23,482 transcripts), cellular component (23,075 transcripts), and molecular function (23,334 transcripts). Cellular process, single organism process, and metabolic process were the most abundant sub-categories of the identified biological processes. Cell, cell part, organelle, and organelle part were the most abundant sub-categories of cellular components. Binding and catalytic activity were the most abundant sub-categories of molecular functions (Figure 3).

### 2.3. High-Level Annotation of Transcripts

Pfam protein domain prediction, isoform coding sequence (CDS) prediction, SMART protein domain prediction, protein properties, and various post-translational modification site predictions were included in the advanced functional annotation information for isoforms. The CDS of a gene is a singular section of DNA or RNA that encodes the corresponding protein. In total, 24,241 transcripts were predicted in the 5′UTR, 25,828 in the 3′UTR, and 26,316 in the CDS, for a total length of 80,018,783. Additionally, we predicted the annotation information related to isoform-encoded protein domains, the prediction results of TMHMM transmembrane helix structure, signal peptide cleavage sites and their location, protein O-GlcNAc glycosylation sites and ProP furin cleavage and more (File S1).

#### 2.3.1. Transcription Factor (TF) Analysis

**Figure 4:**
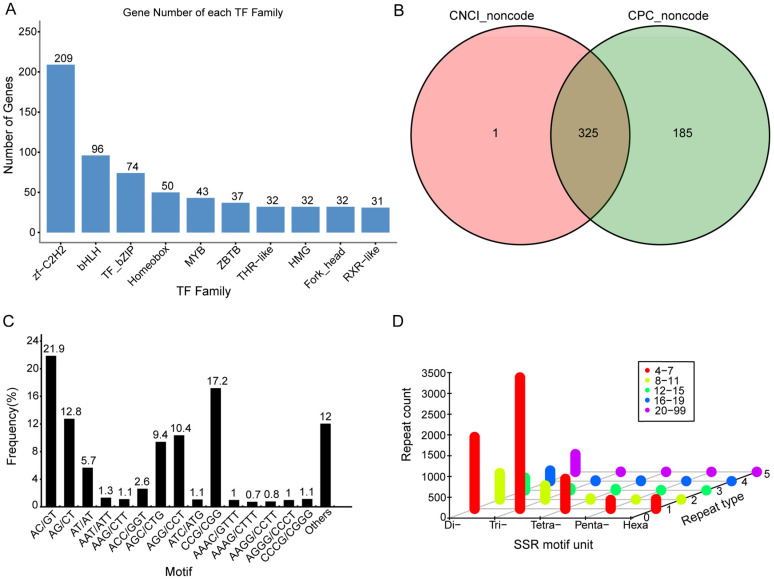
Results of structural analysis for *M. pilosus*. (**A**) The number and family of TFs were predicted by SMRT. (**B**) Venn diagram of lncRNAs predicted by CNCI and CPC methods. (**C**) The proportion of SSRs of different tandem repeat unit types in total SSRs. (**D**) Distribution of SSR types.

Transcription factors (TFs) are key components of gene expression in mammals. TFs are proteins that recognize and bind specific nucleotide sequences during transcription, allowing them to regulate the main expression of nearby genes. In the investigation, DIAMOND software was used to identify 1032 TFs from 59 TF families. The zf-C2H2 family (209) was the most prevalent among the top 10 TF families in (Figure 4A), followed by the BHLH family (96).

#### 2.3.2. Identification of Long Non-Coding RNAs

LncRNAs are non-protein-coding transcripts longer than 200 nucleotides. They participate in biological processes such as transcription, translation, protein localization, cellular structural integrity, reprogramming, and other cellular functions. Altogether, 325 unigenes were identified as lncRNAs in this work (Figure 4B).

#### 2.3.3. SSR Prediction

An SSR is a repetitive DNA sequence in which certain motifs are repeated. Upon scrutiny of the obtained SSRs, the proportions of SSRs of different tandem repeat unit types in total SSR were measured (Figure 4C). The most predominant was the tri-nucleotide repeats (3352), followed by the di-nucleotide repeats (3580) and tetra-nucleotide repeats (800). Penta- and hexa-nucleotide repeats, at 228 and 239, respectively, constituted a relatively minor portion of the total. Additionally, the majority of SSRs had 4–7 repeats (6059) (Figure 4D).

### 2.4. Alternative Splicing Events

**Figure 5:**
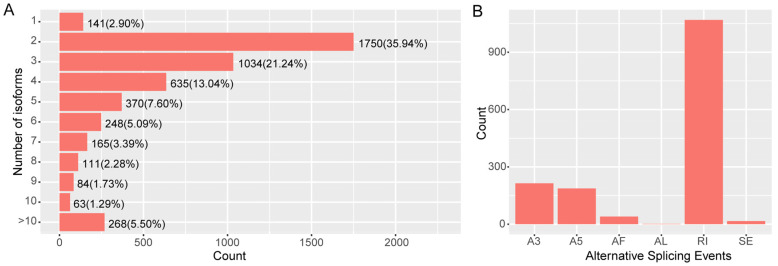
AS events in *M. pilosus.* (**A**) The statistics of isoform number. (**B**) The statistics of AS events.

The analysis identified 1528 alternative splicing events (AS events) of six different types, the most prominent of which were retained introns (RI, 1069, 69.96%), followed by 3′ splice sites (A3, 214, 14%) and 5′ splice sites (A5, 187, 12.23%). A base for the research of alternative splicing in the *M. pilosus* was provided by the discovery that 4869 isoforms had AS events, of which 4728 isoforms contained more than one event (Figure 5). We sequenced a variety of different tissues in order to more comprehensively detect AS events. There needed to be more research completed on the functions of isoforms because AS events may play various roles in various tissues.

### 2.5. Validation of the Full-Length Transcriptome of M. pilosus

Several published genes expressed in *M. pilosus* were compared with the full-length transcripts of bats obtained in this study to verify the accuracy of the sequencing results. The results showed that PacBio sequencing of bats was able to capture the whole length of all genes evaluated, despite some proteins having one or two amino acid mismatches. The comparison of the *PRL-2*, *DPP4*, *Glul*, and *ND1* genes is displayed in Figures S4–S7.

### 2.6. Identification of M. pilosus Transcripts

#### 2.6.1. Identification of Neural-Related Transcripts in *M. pilosus*

According to the GO annotation findings, 409 transcripts were annotated to the presynaptic process involved in synaptic transmission (GO: 0099531) in the biological process category. In addition, 2214 transcripts were annotated to synapse in the cellular component and 1797 transcripts were annotated to the synapse part (GO: 0044456) in the cellular component (Figure 3). Moreover, a total of 2313 genes associated with 10 neural-related pathways were enriched in KEGG pathways. Retrograde endocannabinoid signaling (331 genes), dopaminergic synapse (298 genes), glutamatergic synapse (285 genes), GABAergic synapse (274 genes), neurotrophin signaling pathway (251 genes), and serotonergic synapse (236 genes), were the pathways related to the largest numbers of genes (Figure S8).

#### 2.6.2. Identification of Metabolite-Related Transcripts in *M. pilosus*

In the GO and KEGG databases, several transcripts were annotated in metabolite-related pathways. There were 19,294 transcripts in all that had metabolic process annotations (GO: 0008152), and 10,237 genes in total were enriched in 12 pathways associated with metabolism (Figure 2D). Pathways associated with the higher number of genes were global and overview maps (4183), amino acid metabolism (1316), carbohydrate metabolism (1151), energy metabolism (491), glycan biosynthesis and metabolism (419), lipid metabolism (1268), and metabolism of cofactors and vitamins (668).

#### 2.6.3. Transcript Identification Associated with Auditory Perception in *M. pilosus*

Several genes related to bat high-frequency hearing were identified in the analysis, including *SK2*, *Myo6*, *ACTB*, *FoxP2*, *OTOF*, *Cx43*, *Shh*, *KCNQ4*, *GJB6*, and *SLC26A5*. Despite that some proteins had some amino acid mismatches, the results revealed that all previously published auditory perception genes were detected. Fortunately, the genes *GJB6* (Figure S9) and *SK2* (Figure S10) were successfully sequenced using PacBio sequencing to obtain the full length. In addition, 3927 transcripts were annotated to homeostatic process (GO: 0042592), and 393 transcripts were annotated to tissue homeostasis (GO: 0001894); 499 transcripts were annotated to ossification (GO: 0001503), and 1787 transcripts were annotated to organ morphogenesis (GO: 0009887). There were 670 transcripts annotated to multicellular organismal homeostasis (GO: 0048871). In the KEGG annotation results (Figure 2D), 1208 transcripts were annotated to nervous system pathways and 3274 transcripts were annotated to signal transduction. In terms of function, both of these pathways are closely related to auditory perception. In signal transduction, 132 and 469 genes were annotations of the NF-Kappa B signaling pathway (KO04064) and MAPK signaling pathway (KO04010) that are related to protecting the inner ears of bats from auditory damage caused by long time high-frequency calling.

#### 2.6.4. Identification of Immune-Related Transcripts in *M. pilosus*

**Figure 6:**
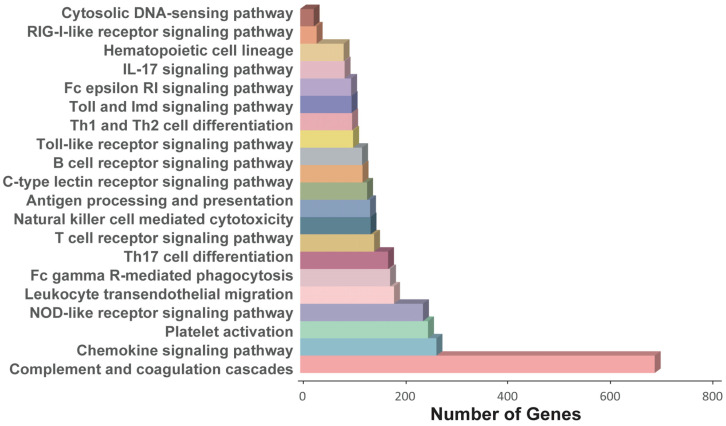
The proportion of full-length transcripts annotated in the metabolic pathways of the immune system.

We associated the gene set with GO terms based on the human-specific gene ontology annotation. There were 4702 immune-related transcripts; among the most represented GO terms were immune response and regulation of immune system process. Due to the unique immune regulation system of bats, many transcripts were annotated in the KEGG database for immune-related pathways. Moreover, we also found that the top five immune pathways with the largest numbers of transcripts among the 21 immune pathways did not have overlapped transcripts (Figure 6 and Figure S11). Pathways associated with higher numbers of genes were complement and coagulation cascades (710 genes) followed by chemokine signaling pathway (273 genes), platelet activation (256 genes), NOD-like receptor-signaling pathway (246 genes), Leukocyte transendothelial migration (188 genes), Fc gamma R-mediated phagocytosis (180 genes), Th17 cell differentiation (176 genes), and T cell receptor signaling pathway (148 genes). These results roughly described the pathways related to immune function.

We checked the transcriptome annotation for the existence of anti-viral genes. A multitude of pattern recognition receptors was identified, including NLR, RLR, SRCR, the antiviral molecule IRF3, and the IFN receptor subunit IFNAR1 (Table 2). Additionally, we examined genes linked to B cells and discovered transcripts for CD27 (Table 2). Finally, we looked at the cytokine TGF and various immunoglobulin domains, which are crucial for determining innate and adaptive immune responses.

## 3. Discussion

During the past decade, transcriptome analysis has proven to be an effective means of gene discovery, genome annotation, and in-depth exploration of genes associated with phenotypic traits. The NGS RNA-Seq transcriptome databases of *R. sinicus* [11], *Rousettus aegyptiacus* [12], and *Vespertilio sinensis* [5] have been reported previously. However, the number of transcript isoforms or the length of the transcript limited these studies. With the development of sequencing techniques, PacBio Iso-Seq, a full-length transcriptome sequencing method that is especially ideal for the direct generation of complete transcriptomes with precise AS isoforms in non-model organisms without genomic sequences, has ushered in a new age of transcriptome-wide study. To provide effective reference genomic information at the transcriptome level for the study of *M. pilosus*, providing a method for the study of bats as well as other organisms regarding the genome is necessary. In the study of the molecular evolution of high-frequency hearing in echolocation bats [13], 130,381 unigenes in the reference gene set of three bats were annotated by second-generation no-reference transcriptomic sequencing. A total of 67,664 unigenes were obtained with annotation information from at least one database, for an annotation rate of 52%. Similarly, 12,427 and 13,539 transcripts were annotated with at least one GO functional category in the second generation of unreferenced transcriptome studies of the ear of *M. pilosus* and *Cynopterus sphinx* [6], with annotation rates of 38% and 30%, respectively. The annotation rate of transcripts obtained in this study was as high as 98%, much higher than the annotation rate obtained by using the previous second-generation sequencing technology. This is consistent with the results of Wan [14] demonstrating that full-length transcriptome sequencing has a higher annotation rate than second-generation sequencing technology. Furthermore, the result indicates that full-length transcriptome sequencing will produce better annotation results.

Previous studies have confirmed the reliability of the PacBio Iso-Seq platform [15], but the accuracy of full-length data can be further improved through a second-generation data correction [16]. In the present work, 1249 low-quality sequences from the bats’ brain area were corrected using PacBio sequencing technology and Illumina sequencing data. A total of 714 low-quality sequences were corrected, the correction rate was 57%; the average length of corrected transcripts was 3027.42 bp and the length of N50 length was 3449 bp. The combined approach of PacBio Iso-Seq and Illumina HiSeq improved the accuracy of the sequencing results and provided a high-confidence transcriptome map for *M. pilosus*, which can be used as a necessary genetic background for basic biological research in bats.

In this work, sophisticated annotations including isoform CDS prediction, Pfam protein domain prediction, SMART protein domain prediction, protein properties, and others are supplied in addition to basic annotations such NR, KEGG, KOG, Swiss-Prot, and GO. Understanding the biological functions of specific proteins expressed in bats and recognizing protein–protein interactions are made possible by studying protein structures. These processes are crucial for the study of adaptive evolution in bats as well as the distinctive immune adaptations in bats. High-level annotation of transcripts is especially necessary for the absence of a genome for *M. pilosus*.

Alternative splicing is a meaningful way to regulate gene expression and it plays a vital role in various biological processes in bats. Elena O. Gracheva et al. [17] confirmed that alternative splicing of TRPV1 transcripts is the basis of infrared perception in vampire bats. Chen [18] studied the role of AS events in response to cellular stress caused by a mitotic mismatch in natural populations of *Rhinolophus affinis*. However, it is exceedingly challenging to examine its AS isoforms owing to the dearth of genetic data. Due to the long length, PacBio sequencing technology can obtain more complete transcripts, that provide a basis for studying AS of *M. pilosus*.

Several published genes expressed in *M. pilosus* (*GJB6*, *Glul*, *ND1*, *DPP4*) were compared with the full-length transcripts of bats obtained in this study to verify the accuracy of the sequencing results. The PacBio sequencing could accurately obtain the full length of the detected genes, indicating that the full-length transcriptome in this study was relatively complete and reliable. The same validation method was applied in the PacBio sequencing of *Heliocidaris crassispina* [19] to verify the accuracy of the sequencing results. The accuracy of the sequencing results also showed that the data could, to a certain extent, be utilized as the genome data of *M. pilosus*, a result that greatly made up for the absence of the genome data of bats.

In this study, transcripts related to bat high-frequency hearing were identified, and homeostatic process (GO: 0042592), tissue homeostasis (GO: 0001894), ossification (GO: 0001503), organ morphogenesis (GO: 0009887) and multicellular organismal homeostasis (GO: 0048871) were found. These physiological processes are related to the adaptive evolution of echolocation and high-frequency hearing in laryngeal echolocation bats [20], but more transcripts directly related to auditory perception were not detected, possibly due to the specificity of cochlear tissue resulting in relatively few genes detected in the cochlea. Ma et al. [10]. reported a relatively low quality of transcriptome assembly in the cochlea due to the highly specialized cochlear tissue, and the same results were also reported in a single-cell RNA-seq study of the mouse cochlea [21]. However, all previously published auditory perception genes were detected, including *SLC26A5*, *SK2*, *Myo6*, *OTOF*, *Shh,* and *KCNQ4*. The remarkable high-frequency sensitivity and selectivity of the bat auditory system have been attributed to the evolution of mechanical amplification, in which sound waves are amplified by outer hair cells in the cochlea. *SK2* is an important ion channel protein-encoding gene that plays an important role in the physiological process of cochlear hearing. These are crucial genes for understanding how echolocating high-frequency auditory adaptations have evolved, and it is believed that they have had a significant influence on the evolution of echolocation [13,22]. The CDS sequences of these genes were also identified in the transcripts, providing a basis for studying the process of auditory perception and adaptive evolution in bats.

Bats are increasingly being considered as potential reservoirs harboring a diverse and complex microbial community, including many known and unknown viruses [23,24,25,26]. Infected bats showed no or minimal signs of disease even when high viral titers were detected in tissues or sera, suggesting that the host defense–immune tolerance balance of bats confers exceptional health. As a result, we searched immune-related transcripts in the transcriptome, and we found several pattern recognition receptors. Altogether, the reference transcriptome generated for *M. pilosus* provides an excellent foundation for investigating reservoir host immunology in bats. However, a comprehensive understanding of its immune system and related immunoglobulin gene repertoire requires further analysis of the genome.

## 4. Materials and Methods

### 4.1. Sample Collection

Two bats were caught from a colony with more than 1500 individuals that roosted in a cave in Beijing, China (115°59′ N, 39°43′ E). The bats that returned to their roost site after predation were captured by mist nets and were then caught from the mist nets with sterile gloves. Each captured individual was put into a sterilized cloth bag. Bats were rapidly euthanized by cervical dislocation, and five tissues, the cochlea, brain, muscle, liver, and heart, were collected and transferred to RNase-free PCR tubes. Tissue samples were frozen immediately in liquid nitrogen and stored at −80 °C until RNA extraction.

### 4.2. Library Construction and SMRT Sequencing

Total RNA from bat tissue was extracted using TRIzol reagent (Life Technologies, Carlsbad, CA, USA) on dry ice following the standard protocol provided by the manufacturer. The RNA concentration and purity were assessed by an Agilent 2100 Bioanalyzer (Agilent Technologies, Santa Clara, CA, USA), agarose gel electrophoresis, and a Nanodrop micro-spectrophotometer (Thermo Fisher, Waltham, MA, USA) to verify RNA integrity. The mRNA was enriched using oligo dT magnetic beads. Then, the enriched mRNA was reverse transcribed into cDNA using a Clontech SMARTer PCR cDNA Synthesis Kit (Takara Bio Inc., Kusatsu, Japan). Primers, polymerases, and magnetic beads were loaded to generate a completed SMRT bell library, and sequencing of the library was performed on the PacBio RS II (Pacific Biosciences, Menlo Park, CA, USA) platform. The raw sequencing reads of cDNA libraries were analyzed by using an isoform sequencing (Iso-Seq) pipeline supported by Pacific Biosciences [27].

### 4.3. Data Processing and Error Correction of PacBio Iso-Seq Reads

Using the second-generation Illumina sequencing data obtained from brain tissues of three individual bats that were sampled at the same time and location, low-quality sequences were corrected for LoRDEC using a hybrid strategy that required the use of two sets of data: reference reads (second-generation short reads), and PacBio long reads. De Bruijn plots were created by reading the short reads and searching for erroneous sequences in the long reads to find the correction sequence for the error region in the long reads. After correction, we took the low-quality sequences with correction coverage (percentage of bases corrected in the second-generation data to the third-generation consistent sequences) of 99% or more and merged them with the high-quality sequences obtained by Quiver correction to obtain more accurate transcripts for subsequent analysis.

### 4.4. Functional Annotation of Transcripts

Basic annotation of isoforms includes protein functional annotation, pathway annotation, KOG functional annotation, and Gene Ontology (GO) annotation. Isoforms were then annotated using the NCBI non-redundant protein (Nr) database (http://www.ncbi.nlm.nih.gov, accessed on 16 November 2021), the Swiss-Prot protein database (http://www.expasy.ch/sprot, accessed on 20 November 2021), the Kyoto Encyclopedia of Genes and Genomes (KEGG) database (http://www.genome.jp/kegg, accessed on 20 November 2021), and the KOG database (http://www.ncbi.nlm.nih.gov/COG, accessed on 22 November 2021) with BLASTx program (http://www.ncbi.nlm.nih.gov/BLAST/, accessed on 22 November 2021). Gene Ontology (GO) annotation was conducted using the blast2GO [28] program with Nr annotation results of isoforms. Isoforms ranking in the top 20 highest scores and having no fewer than 33 HSPs (high-scoring segment pair) hits were selected to conduct functional classification. Functional classification of isoforms was performed using WEGO 1.0 software [29].

### 4.5. Open Reading Frame Prediction and Protein Domain Prediction

The coding sequence (CDS), protein sequence, and UTR sequence were obtained, and the isoform sequence of the open reading frame (ORF) was detected by ANGEL [30] software. Protein domain prediction was performed by aligning protein sequences of isoforms to the Pfam database (version 26.0) by Pfam Scan program [31] and SMART database (version 7.0) by HMMER—profile hidden Markov models for biosequence analysis program (http://hmmer.org/, accessed on 30 November 2021) to obtain protein domain annotations.

### 4.6. Transcription Factor (TF) Analysis and Potential Protein Function Analysis

For bats, protein-coding sequences of isoforms were aligned by hmmscan to Animal TFdb (http://www.bioguo.org/AnimalTFDB/, accessed on 2 December 2021) to predict TF families.

Potential transmembrane helices in proteins were predicted by TMHMM Server 2.0 and SignalP 4.1 Server software, and the presence and location of signal peptide cleavage sites in amino acid sequences were predicted. Mucin-type GalNAc O-glycosylation sites in mammalian proteins and arginine and lysine propeptide cleavage sites in eukaryotic protein sequences were predicted by the NetOGlyc 4.0 Server and the ProP 1.0 Server.

### 4.7. Simple Sequence Repeats (SSR) Prediction

The MIcroSAtellite (MISA, http://pgrc.ipk-gatersleben.de/misa/, accessed on 8 December 2021) was employed for microsatellite mining in the whole transcriptome. The parameters were as follows:

Definition (unit size, min repeats): 2–6 3–5 4–4 5–4 6–4

Interruptions (max difference between two SSRs): 100

Notes:

(1) (n, m): n is the length of repeat units. m is the minimum number of repeat units.
(2) Interruptions: the distance between two SSRs less than 100 bp is considered as one SSR

### 4.8. Characterization of Long Non-Coding RNAs and Alternative Splicing Detection

CNCI (version 2) [32] was used to assess the protein-coding potential of transcripts without annotations using default parameters for potential long non-coding RNAs. To focus on relatively reliable lncRNA annotations at evolution level, the software Infernal 1.1 [33] (http://eddylab.org/infernal/, accessed on 8 December 2021) was used in sequence alignment. Classification of lncRNAs is based on secondary structures and sequence conservation. To analyze alternative splicing events of transcript isoforms, COding GENome reconstruction Tool (Cogent) [34] was first used to partition transcripts into gene families based on k-mer similarity and to reconstruct each family into a coding reference genome based on De Bruijn graph methods. Alternative splicing events of transcript isoforms were performed using SUPPA tool [35].

### 4.9. Validating the Accuracy of Sequencing Result

In order to validate the accuracy of the sequencing results, we aligned the published gene sequences of *M. pilosus* with Isoform sequences. Several genes were identified by referring to the relevant papers concerning *M. pilosus*, including hearing-related genes *GJB6* (GenBank: GU326374.1) [36] and *SK2* (GenBank: MT822709.1), the immune-related gene *DPP4* (GenBank: MH345673.1) [37], and metabolism-related genes *Glul* (GenBank: KJ561605.1), *ND1* (GenBank: AB106586.1) [38], and *PRL-2* (GenBank: EF534354.1) [39]. For comparison with the isoform sequences according to the GeneBank numbers in published papers, the whole CDS sequences were downloaded from the NCBI database. The downloaded nucleic acid sequence and the isoform sequence were then translated into amino acid sequences, and the two translations were then compared at the protein level. The same method was used to compare *Myo6* (GenBank: JX023452.1), *ACTB* (GenBank: JQ284421.1), *FoxP2* (GenBank: EU076403.1), *OTOF* (GenBank: JQ284410.1), *Cx43* (GenBank: EU195813.1), *Shh* (GenBank: KX495654.1), *KCNQ4* (GenBank: HE608291.1), and *SLC26A5* (GenBank: EU914924.1), all of which were associated with high-frequency hearing, in previously published studies on *M. pilosus.*

### 4.10. Identification of Immune-Related Genes

Several immune-related genes and pattern recognition receptors were identified using the Hidden Markov Model (HMM) from the Pfam database (http://pfam.xfam.org/, accessed on 9 December 2021), including NLR (PF05729, NACHT domain), RLR (PF11648, RIG-I domain), SRCR (PF00530, scavenger receptor cysteine-rich domain), the IFN receptor subunit IFNAR1, antiviral molecule IRF-3 and genes associated with adaptive immune response and B cells, as well as cytokines and chemokines crucial for the immune response, both innate and adaptive. Afterward, all members were verified using the HMMER database. We next searched for specific genes related to various aspects of the immune responses in other mammals, primarily mice and humans.

## 5. Conclusions

The first full-length transcriptome of *M. pilosus* was constructed herein using both Illumina HiSeq and PacBio sequencing platforms. Gene annotation, CDS prediction, transcription factors, SSR discovery, lncRNA prediction, and AS analysis were performed without the reference genome. A number of transcripts related to auditory perception and immunity were identified. These findings were helpful in identifying different types of alternative splicing in *M. pilosus*. They can also be used to compare bat genomes and identify the genetic changes that may have influenced bat evolution. The related functional genes discovered in the study will serve as a guide for further research into the molecular basis of bats’ unique immunity, as well as human aging and molecular disease targets.

## Figures and Tables

**Table 1:** Description of the transcriptome of *M. pilosus* by PacBio Iso-Seq.

Sample	*M. pilosus*
Subreads number	17,570,601
Mean length (bp)	2622
CCS number	752,249
Mean CCS read length (bp)	2978
Number of transcripts	29,456
Number of non-redundant transcripts	26,717
Mean length (bp)	3018.91

**Table 2:** NACHT, RIG-I, IFN, IRF-3, CD20, and TGFβ domain containing proteins of the full-length transcriptome of *M. pilosus*.

Isoform	Structure
Isoform0026163	NACHT
Isoform0007843	NACHT
Isoform0007306	NA
Isoform0006981	RIG-I_C-RD
Isoform0008035	RIG-I_C-RD
Isoform0015581	RIG-I_C-RD
Isoform0017682	IFNGR1
Isoform0019310	IFNGR1
Isoform0022891	IRF-3
Isoform0023355	IRF-3
Isoform0006241	IRF-3
Isoform0011995	IRF-3
Isoform0018495	IRF-3
Isoform0019133	IRF-3
Isoform0019959	IRF-3
Isoform0022054	CD20
Isoform0022520	CD20
Isoform0024768	CD20
Isoform0010536	CD20
Isoform0015085	CD20
Isoform0019840	CD20
Isoform0021250	CD20
Isoform0021512	CD20
Isoform0025649	Cd27 binding protein (Siva)
Isoform0000889	TGF_beta_GS
Isoform0026593	TGF_beta_GS
Isoform0001938	TGFb_propeptide
Isoform0001938	TGF_beta
Isoform0003427	TGF_beta_GS
Isoform0009838	TGF_beta_GS
Isoform0012071	TGF_beta_GS
Isoform0011693	TGF_beta
Isoform0014684	TGFb_propeptide
Isoform0014684	TGF_beta
Isoform0012071	TGF_beta_GS
Isoform0014741	TGFb_propeptide
Isoform0014741	TGF_beta
Isoform0016251	TGFb_propeptide
Isoform0016251	TGF_beta
Isoform0017557	TGFb_propeptide
Isoform0017557	TGF_beta
Isoform0010407	TGF_beta_GS
Isoform0011693	TGFb_propeptide

## Data Availability

The raw sequence data and the transcripts generated were deposited into the NCBI SRA database (accession number: SRR22318483) and the TSA database (accession number: GKEA01000000), respectively.

## Supplementary Materials

The following supporting information can be downloaded at: https://www.mdpi.com/article/10.3390/ijms24010062/s1.
