# Supplementary material for: Full-Length Transcriptome of Myotis pilosus as a Reference Resource and Mining of Auditory and Immune Related Genes
Source: Int J Mol Sci. 2022 Dec 21;24(1):62. doi: 10.3390/ijms24010062 (PMC9820712; doi:10.3390/ijms24010062)
Supplement: Supplementary file 1 [file ijms-24-00062-s001.zip › ijms-2105407-supplementary/supplementary figures.pdf]

Supplemental materials

Figure S1. Tissue sample PacBio sequencing results.

Figure S2. Benchmarking universal single-copy orthologs (BUSCO) assessment results of the transcriptome generated.

Figure S3. The mean lengths of the subreads, CCS and transcripts in *R. a. hainanus*, *R. a. himalayanus* and *M. Pilosus*.

Figure S4. The comparative result of the *PRL-2* gene.

Figure S5. Several published genes were compared with full-length transcripts. The figure shows the comparative results for the *DPP4* gene.

Figure S6. Several published genes were compared with full-length transcripts. The comparative result of the *Glul* gene.

Figure S7. Several published genes were compared with full-length transcripts. The figure shows the comparative results for the *ND1* gene**.**

Figure S8. The proportion of full-length transcripts annotated in the metabolic pathways of the neural-related system.

Figure S9. Several published genes were compared with full-length transcripts. The figure shows the comparative results for the *GJB6* gene.

Figure S10. Several published genes were compared with full-length transcripts. The figure shows the comparative results for the *SK2* gene.

Figure S11. The number of overlapped transcripts among the top five immune pathways with the largest number of genes in the KEGG annotation.

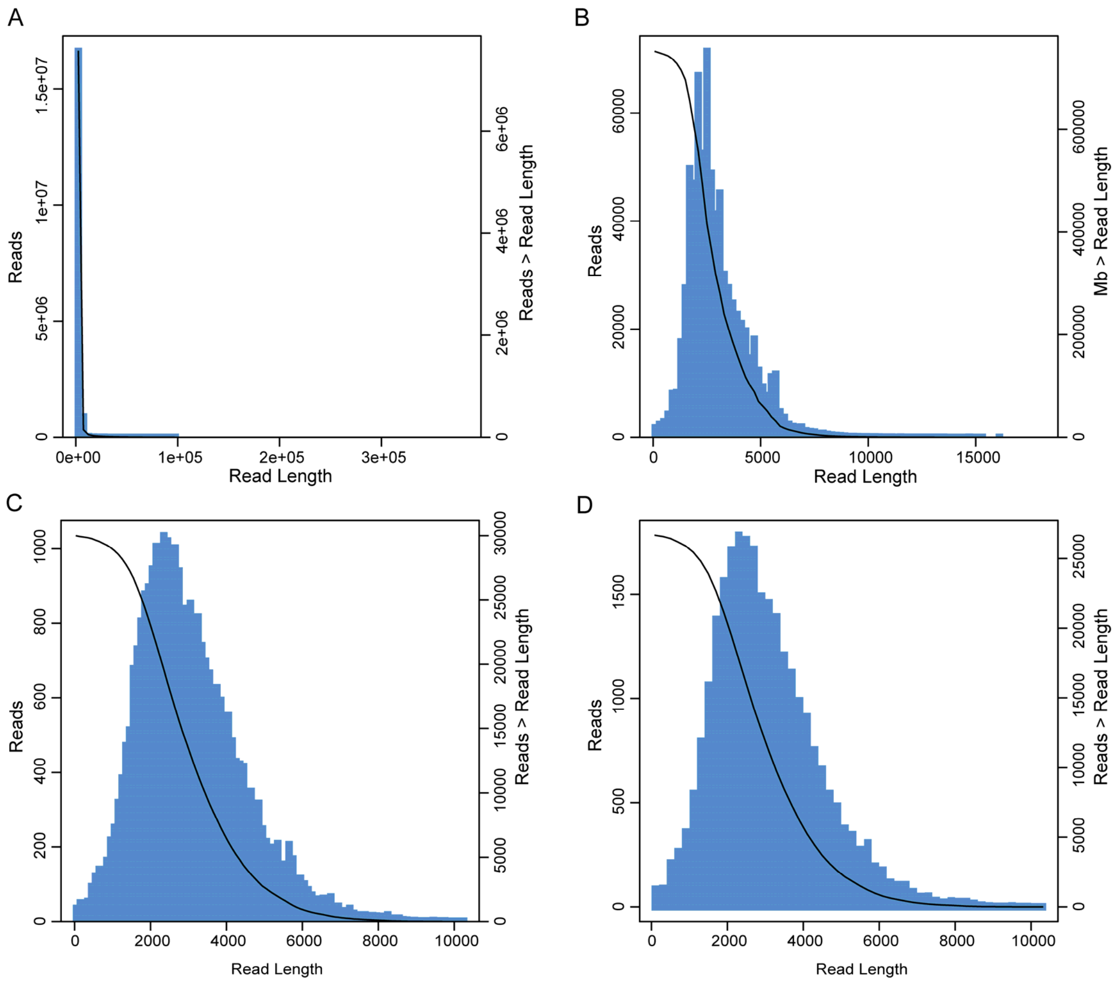

Figure S1 Tissue sample PacBio sequencing results. (A) Length distribution of subreads **(B)**Length distribution of CCS **(C)**Length distribution of Unpolished Consensus Isoforms **(D)** Length distribution of Isoform

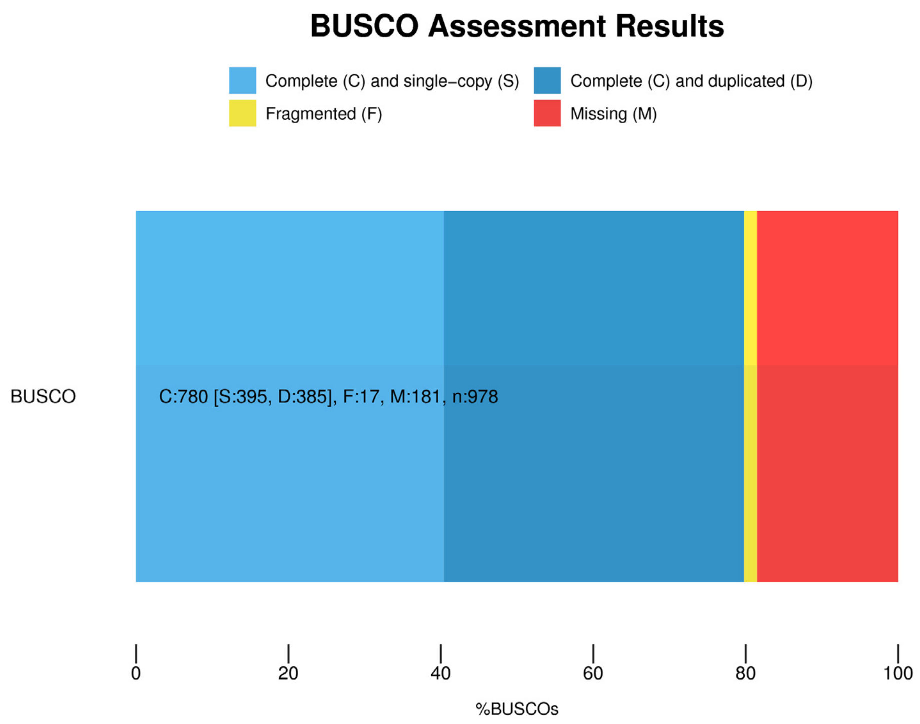

Figure S2. Benchmarking universal single-copy orthologs (BUSCO) assessment results of transcriptome generated.

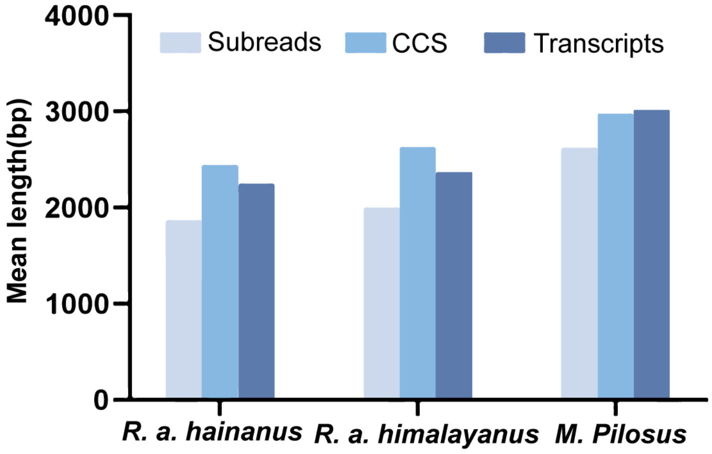

Figure S3. The mean length of the subreads, CCS and transcripts in *R. a. hainanus*, *R. a. himalayanus* and *M. Pilosus*, respectively.

```
Isoform0022110    TTTGCCATAATGAACCGTCCAGCCCCTGTGGAGATCTCCT     40
PRL-2             .........ATGAACCGTCCAGCCCCTGTGGAGATCTCCT     31
Consensus                  atgaaccgtccagcccctgtggagatctcct

Isoform0022110    ATGAGAACATGCGTTTTCTGATTACTCACAACCCTACCAA     80
PRL-2             ATGAGAACATGCGTTTTCTGATTACTCACAACCCTACCAA     71
Consensus         atgagaacatgcgttttctgattactcacaaccctaccaa

Isoform0022110    TGCTACCCTCAACAAGTTCACAGAGGAACTTAAGAAGTAT    120
PRL-2             TGCTACCCTCAACAAGTTCACAGAGGAACTTAAGAAGTAT    111
Consensus         tgctaccctcaacaagttcacagaggaacttaagaagtat

Isoform0022110    GGAGTGACAACTTTGGTTCGAGTTTGTGATGCTACATACG    160
PRL-2             GGAGTGACAACTTTGGTTCGAGTTTGTGATGCTACATACG    151
Consensus         ggagtgacaactttggttcgagtttgtgatgctacatacg

Isoform0022110    ATAAAGCTCCAGTTGAAAAAGAAGGAATCCATGTTCTAGA    200
PRL-2             ATAAAGCTCCAGTTGAAAAAGAAGGAATCCATGTTCTAGA    191
Consensus         ataaagctccagttgaaaaagaaggaatccatgttctaga

Isoform0022110    TTGGCCATTTGATGATGGTGCGCCACCCCCTAATCAGATA    240
PRL-2             TTGGCCATTTGATGATGGTGCGCCACCCCCTAATCAGATA    231
Consensus         ttggccatttgatgatggtgcgccaccccctaatcagata

Isoform0022110    GTGGACGATTGGCTAAACCTATTAAAAACCAAATTTCGTG    280
PRL-2             GTGGACGATTGGCTAAACCTATTAAAAACCAAATTTCGTG    271
Consensus         gtggacgattggctaaacctattaaaaaccaaatttcgtg

Isoform0022110    AAGAACCAGGTTGCTGTGTTGCAGTGCATTGTGTTGCGGG    320
PRL-2             AAGAACCAGGTTGCTGTGTTGCAGTGCATTGTGTTGCGGG    311
Consensus         aagaaccaggttgctgtgttgcagtgcattgtgttgcggg

Isoform0022110    ATTGGGAAGGGCACCTGTGCTGGTTGCACTTGCTTTGATT    360
PRL-2             ATTGGGAAGGGCACCTGTGCTGGTTGCACTTGCTTTGATT    351
Consensus         attgggaagggcacctgtgctggttgcacttgctttgatt

Isoform0022110    GAATGTGGAATGAAGTATGAAGATGCAGTTCAGTTTATAA    400
PRL-2             GAATGTGGAATGAAGTATGAAGATGCAGTTCAGTTTATAA    391
Consensus         gaatgtggaatgaagtatgaagatgcagttcagtttataa

Isoform0022110    GACAAAAAAGAAGGGGAGCATTCAATTCCAAACAGCTGCT    440
PRL-2             GACAAAAAAGAAGGGGAGCATTCAATTCCAAACAGCTGCT    431
Consensus         gacaaaaaagaaggggagcattcaattccaaacagctgct

Isoform0022110    TTACCTGGAGAAATACCGACCTAAGATGCGATTACGCTTC    480
PRL-2             TTACCTGGAGAAATACCGACCTAAGATGCGATTACGCTTC    471
Consensus         ttacctggagaaataccgacctaagatgcgattacgcttc

Isoform0022110    AGAGATACCAATGGGCATTGCTGTGTTCAG..            510
PRL-2             AGAGATACCAATGGGCATTGCTGTGTTCAGTA            503
Consensus         agagataccaatgggcattgctgtgttcag
```

```
prl-2               ...MNRPAPVEISYENMRFLITHNPTNATLNKFTEELKKY     37
Isoform0022110      FAIMNRPAPVEISYENMRFLITHNPTNATLNKFTEELKKY     40
Consensus              mnrpapveisyenmrflithnptnatlnkfteelkky

prl-2               GVTTLVRVCDATYDKAPVEKEGIHVLDWPFDDGAPPPNQI     77
Isoform0022110      GVTTLVRVCDATYDKAPVEKEGIHVLDWPFDDGAPPPNQI     80
Consensus           gvttlvrvcdatydkapvekegihvldwpfddgapppnqi

prl-2               VDDWLNLLKTKFREEPGCCVAVHCVAGLGRAPVLVALALI    117
Isoform0022110      VDDWLNLLKTKFREEPGCCVAVHCVAGLGRAPVLVALALI    120
Consensus           vddwlnllktkfreepgccvavhcvaglgrapvlvalali

prl-2               ECGMKYEDAVQFIRQKRRGAFNSKQLLYLEKYRPKMRLRF    157
Isoform0022110      ECGMKYEDAVQFIRQKRRGAFNSKQLLYLEKYRPKMRLRF    160
Consensus           ecgmkyedavqfirqkrrgafnskqllylekyrpkmrlrf

prl-2               RDTNGHCCV                                   166
Isoform0022110      RDTNGHCCV                                   169
Consensus           rdtnghccv
```

Figure S4

Several published genes were compared with full-length transcripts, the comparative result of the *PRL-2* gene.

```
Isoform0010147    ATGAAGACGCAATGGAAGGTGCTGCTGGGACTGCTGGGGG     40
DPP4              ATGAAGACGCAATGGAAGGTGCTGCTGGGACTGCTGGGGG     40
Consensus         atgaagacgcaatggaaggtgctgctgggactgctggggg

Isoform0010147    CTGCGGCGCTCGTCACCATCATCACCGTGCCCGCGGTTCT     80
DPP4              CTGTGGCGCTCGTCACCATCATCACCGTGCCAGCGGTTCT     80
Consensus         ctg ggcgctcgtcaccatcatcaccgtgcc gcggttct

Isoform0010147    GCTGAGCAAAGATGATGTTATAGTTGATGATCGCAGGACC    120
DPP4              GCTGAGCAAAGATGATGTTATAGTTGATGATCGCAGGACC    120
Consensus         gctgagcaaagatgatgttatagttgatgatcgcaggacc

Isoform0010147    TACACTCTAGCTGATTATTTAAAAAGTACTATTAGAATGA    160
DPP4              TACACTCTAGCTGATTATTTAAAAAGTACTATTAGAATGA    160
Consensus         tacactctagctgattatttaaaaagtactattagaatga

Isoform0010147    GGAACTACAACTTGCGTTGGATTTCAGACCATGAATATCT    200
DPP4              GGAACTACAACTTGCGTTGGATTTCAGACCATGAATATCT    200
Consensus         ggaactacaacttgcgttggatttcagaccatgaatatct

Isoform0010147    CTACAAACAAGAAAATAATGTCTTGCTATTCAATGCTGAC    240
DPP4              CTACAAACAAGAAAATAATGTCTTGCTATTCAATGCTGAC    240
Consensus         ctacaaacaagaaaataatgtcttgctattcaatgctgac

Isoform0010147    CATGGAAACAGCTCCACTTTCTTGGAGAACAGTACATTTG    280
DPP4              CATGGAAACAGCTCCACTTTCTTGGAGAACAGTACATTTG    280
Consensus         catggaaacagctccactttcttggagaacagtacatttg

Isoform0010147    ATCAATTTGGACATTCTATAAGTGATTATTCAGTGTCTCC    320
DPP4              ATCAATTTGGACATTCTATAAGTGATTATTCAGTGTCTCC    320
Consensus         atcaatttggacattctataagtgattattcagtgtctcc

Isoform0010147    TGATAGACAGTTTGTTCTCTTCGAGTACAACTATGTGAAG    360
DPP4              TGATAGACAGTTTGTTCTCTTCGAGTACAACTATGTGAAG    360
Consensus         tgatagacagtttgttctcttcgagtacaactatgtgaag

Isoform0010147    AAATGGAGACATTCCTACACAGCTTCATATGACATTTATG    400
DPP4              AAATGGAGACATTCCTACACAGCTTCATATGACATTTATG    400
Consensus         aaatggagacattcctacacagcttcatatgacatttatg

Isoform0010147    ACTTAAACAAAAGGCAGCTGATAACAGAAGAAAGAATTCC    440
DPP4              ACTTAAACAAAAGGCAGCTGATAACAGAAGAAAGAATTCC    440
Consensus         acttaaacaaaaggcagctgataacagaagaaagaattcc

Isoform0010147    AAATGACACACAGTTGATCCGATGGTCACCAGAGGGTCAT    480
DPP4              AAATGACACACAGTTGATCCGATGGTCACCAGAGGGTCAT    480
Consensus         aaatgacacacagttgatccgatggtcaccagagggtcat

Isoform0010147    AAACTGGCATATGTTTGGAACAATGACATTTATGTTAAAA    520
DPP4              AAACTGGCATATGTTTGGAACAATGACATTTATGTTAAAA    520
Consensus         aaactggcatatgtttggaacaatgacatttatgttaaaa

Isoform0010147    ATGATCCATATTCACCAAGTCAGAGGGTCACACGTGATGG    560
DPP4              ATGATCCATATTCACCAAGTCAGAGGGTCACACGTGATGG    560
Consensus         atgatccatattcaccaagtcagagggtcacacgtgatgg

Isoform0010147    AAGAGAAGATGCAATCAGTAATGGAATAACTGACTGGGTT    600
DPP4              AAGAGAAGATGCAATCAGTAATGGAATAACTGACTGGGTT    600
Consensus         aagagaagatgcaatcagtaatggaataactgactgggtt

Isoform0010147    TATGAAGAGGAAATCTTCAGTACTCACTCCGCTCTGTGGT    640
DPP4              TATGAAGAGGAAATCTTCAGTACTCACTCCGCTCTGTGGT    640
Consensus         tatgaagaggaaatcttcagtactcactccgctctgtggt

Isoform0010147    GGTCTCCAAACGGCACATTTTTAGCATATGCCCAGTTTAA    680
DPP4              GGTCTCCAAACGGCACATTTTTAGCATATGCCCAGTTTAA    680
Consensus         ggtctccaaacggcacatttttagcatatgcccagtttaa

Isoform0010147    CGACACCGACGTCCCACGCATTGAATACTCTGTCTACTTG    720
DPP4              CGACACCGACGTCCCACGCATTGAATACTCTGTCTACTTG    720
Consensus         cgacaccgacgtcccacgcattgaatactctgtctacttg

Isoform0010147    GACGAGTCGCTGCAGTACCCAAAGACTATCCACATTCCAT    760
DPP4              GACGAGTCGCTGCAGTACCCAAAGACTATCCACATTCCAT    760
Consensus         gacgagtcgctgcagtacccaaagactatccacattccat

Isoform0010147    ACCCAAAGGCAGGAGCTAAGAATCCAACTGTAAAGCTCTA    800
DPP4              ACCCAAAGGCAGGAGCTAAGAATCCAACTGTAAAGCTCTA    800
Consensus         acccaaaggcaggagctaagaatccaactgtaaagctcta

Isoform0010147    TGTTGTAAATACTGACAACCTCACCGATCTAGAGCCTGTG    840
DPP4              TGTTGTAAATACTGACAACCTCACCGATCTAGAGCCTGTG    840
Consensus         tgttgtaaatactgacaacctcaccgatctagagcctgtg

Isoform0010147    CAAATAGTTGCTCCTGCTTCTGTGTTAATAGGGGATCACT    880
DPP4              CAAATAGTTGCTCCTGCTTCTGTGTTAATAGGGGATCACT    880
Consensus         caaatagttgctcctgcttctgtgttaataggggatcact

Isoform0010147    ACTTGTGTGACGTGACATGGGCAACCAAAGAAAGGATTTC    920
DPP4              ACTTGTGTGACGTGACATGGGCAACCAAAGAAAGGATTTC    920
Consensus         acttgtgtgacgtgacatgggcaaccaaagaaaggatttc

Isoform0010147    TTTGCAGTGGCTCCGGAGGATTCAGAACTATTCAATAATA    960
DPP4              TTTGCAGTGGCTCCGGAGGATTCAGAACTATTCAATAATA    960
Consensus         tttgcagtggctccggaggattcagaactattcaataata

Isoform0010147    GATATTTGTGACTATAATGAGTCCACTCCCAAATGGAATT   1000
DPP4              GATATTTGTGACTATAATGAGTCCACTCCCAAATGGAATT   1000
Consensus         gatatttgtgactataatgagtccactcccaaatggaatt

Isoform0010147    GCTTAGTGTCACGGCAACATATTGAAACGAGTGCTACTGG   1040
DPP4              GCTTAGTGTCACGGCAACATATTGAAACGAGTGCTACTGG   1040
Consensus         gcttagtgtcacggcaacatattgaaacgagtgctactgg

Isoform0010147    CTGGGTTGGAAGATTTAAGCCTGCAGAACCTCATTTTACC   1080
DPP4              CTGGGTTGGAAGATTTAAGCCTGCAGAACCTCATTTTACC   1080
Consensus         ctgggttggaagatttaagcctgcagaacctcattttacc

Isoform0010147    TCTGATGGGAATAGCTTCTACAAGATCATGAGCAATACTG   1120
DPP4              TCTGATGGGAATAGCTTCTACAAGATCATGAGCAATACTG   1120
Consensus         tctgatgggaatagcttctacaagatcatgagcaatactg

Isoform0010147    AGGGCTACAAACACATTTGTCTTTTCCAAATAGATAAGCC   1160
DPP4              AGGGCTACAAACACATTTGTCTTTTCCAAATAGATAAGCC   1160
Consensus         agggctacaaacacatttgtcttttccaaatagataagcc

Isoform0010147    AAATTGCACATTTATTACAAAAGGAGCCTGGGAAGTCATC   1200
DPP4              AAATTGCACATTTATTACAAAAGGAGCCTGGGAAGTCATC   1200
Consensus         aaattgcacatttattacaaaaggagcctgggaagtcatc

Isoform0010147    GGGATAGAAGCCCTTACCAACGATTACCTATACTACATCA   1240
DPP4              GGGATAGAAGCCCTTACCAACGATTACCTATACTACATCA   1240
Consensus         gggatagaagcccttaccaacgattacctatactacatca

Isoform0010147    GTAATGAATATAAAGGAATGCCAGGAGGAAGAAATCTTTA   1280
DPP4              GTAATGAATATAAAGGAATGCCAGGAGGAAGAAATCTTTA   1280
Consensus         gtaatgaatataaaggaatgccaggaggaagaaatcttta

Isoform0010147    TAAAATCCAACGTAATAACTACGCAAACGTGACATGTCTG   1320
DPP4              TAAAATCCAACGTAATAACTACGCAAACGTGACATGTCTG   1320
Consensus         taaaatccaacgtaataactacgcaaacgtgacatgtctg

Isoform0010147    AGTTGTGAGCTGGATCCAGAAAGGTGTCAGTACTATTCGG   1360
DPP4              AGTTGTGAGCTGGATCCAGAAAGGTGTCAGTACTATTCGG   1360
Consensus         agttgtgagctggatccagaaaggtgtcagtactattcgg

Isoform0010147    CATCATTTAGTAAAGGGGCGAAATACTATCAGCTGAGATG   1400
DPP4              CATCATTTAGTAAAGGGGCGAAATACTATCAGCTGAGATG   1400
Consensus         catcatttagtaaaggggcgaaatactatcagctgagatg

Isoform0010147    TTCAGGCCCGCAAATCCCCCGCTATTCTCTGCATAGCAGC   1440
DPP4              TTCAGGCCCGCAAATCCCCCGCTATTCTCTGCATAGCAGC   1440
Consensus         ttcaggcccgcaaatcccccgctattctctgcatagcagc

Isoform0010147    AGCAATGATAAAGAACTGAGACTCCTGGAAAACAATACAG   1480
DPP4              AGCAATGATAAAGAACTGAGACTCCTGGAAAACAATACAG   1480
Consensus         agcaatgataaagaactgagactcctggaaaacaatacag

Isoform0010147    CTTTGTATGAGACACTGCAGAACATCCAGATGCCAAGAAA   1520
DPP4              CTTTGTATGAGACACTGCAGAACATCCAGATGCCAAGAAA   1520
Consensus         ctttgtatgagacactgcagaacatccagatgccaagaaa

Isoform0010147    AACACTGGACTTCATTCATCTGAATGGGACAAAATTTTGG   1560
DPP4              AACACTGGACTTCATTCATCTGAATGGGACGAAATTTTGG   1560
Consensus         aacactggacttcattcatctgaatgggac aaattttgg

Isoform0010147    TATCAGATGATCTTGCCTCCTCATTTTGATAAATCCAAGA   1600
DPP4              TATCAGATGATCTTGCCTCCTCATTTTGATAAATCCAAGA   1600
Consensus         tatcagatgatcttgcctcctcattttgataaatccaaga

Isoform0010147    AATATCCTCTACTAATAGATGTGTATGCAGGCCCCTGTAG   1640
DPP4              AATATCCTCTACTAATAGATGTGTATGCAGGCCCCTGTAG   1640
Consensus         aatatcctctactaatagatgtgtatgcaggcccctgtag

Isoform0010147    TCAAAAAGCAGATGCCACCTTCAAACTCAGTTGGGCTACT   1680
DPP4              TCAAAAAGCAGATGCCACCTTCAAACTCAGTTGGGCTACT   1680
Consensus         tcaaaaagcagatgccaccttcaaactcagttgggctact

Isoform0010147    TACCTTGCAAGCACAGAAAATATTATAGTAGCTAGCTTTG   1720
DPP4              TACCTTGCAAGCACAGAAAATATTATAGTAGCTAGCTTTG   1720
Consensus         taccttgcaagcacagaaaatattatagtagctagctttg

Isoform0010147    ATGGCAGAGGAAGTGGTTACCAAGGAGATAAGATCATGCA   1760
DPP4              ATGGCAGAGGAAGTGGTTACCAAGGAGATAAGATCATGCA   1760
Consensus         atggcagaggaagtggttaccaaggagataagatcatgca

Isoform0010147    TGCAATCAACAGAAGATTGGGAACATTTGAAGTTGAAGAT   1800
DPP4              TGCAATCAACAGAAGATTGGGAACATTTGAAGTTGAAGAT   1800
Consensus         tgcaatcaacagaagattgggaacatttgaagttgaagat

Isoform0010147    CAAATTGAAGCAGCCAAACAATTTTCAAAAATGGGATTTG   1840
DPP4              CAAATTGAAGCAGCCAAACAATTTTCTAAAATGGGATTTG   1840
Consensus         caaattgaagcagccaaacaattttc aaaatgggatttg

Isoform0010147    TGGACGACAAACGAATTGCAATTTGGGGCTGGTCATATGG   1880
DPP4              TGGACGACAAACGAATTGCAATTTGGGGCTGGTCATATGG   1880
Consensus         tggacgacaaacgaattgcaatttggggctggtcatatgg

Isoform0010147    AGGGTACGTAACCTCAATGGTCCTGGGAGCAGGAAGTCGT   1920
DPP4              AGGGTACGTAACCTCAATGGTCCTGGGAGCAGGAAGTCGT   1920
Consensus         agggtacgtaacctcaatggtcctgggagcaggaagtcgt

Isoform0010147    GTGTTCAAGTGTGGAATAGCCGTGGCGCCTGTGTCAGCAT   1960
DPP4              GTGTTCAAGTGTGGAATAGCCGTGGCGCCTGTGTCAGCAT   1960
Consensus         gtgttcaagtgtggaatagccgtggcgcctgtgtcagcat

Isoform0010147    GGGAGTTCTATGACTCAGTGTACACAGAACGTTACATGGG   2000
DPP4              GGGAGTTCTATGACTCAGTGTACACAGAACGTTACATGGG   2000
Consensus         gggagttctatgactcagtgtacacagaacgttacatggg

Isoform0010147    TCTCCCTACTCCAGAAGACAACCTTGACCATTACAAGAAT   2040
DPP4              TCTCCCTACTCCAGAAGACAACCTTGACCATTACAAGAAT   2040
Consensus         tctccctactccagaagacaaccttgaccattacaagaat

Isoform0010147    TCAACAGTCATGAGCAGAGCTGAAAATTTTAAACTCGTCG   2080
DPP4              TCAACAGTCATGAGCAGAGCTGAAAATTTTAAACTCGTCG   2080
Consensus         tcaacagtcatgagcagagctgaaaattttaaactcgtcg

Isoform0010147    AGTACCTCCTTATTCATGGAACCGCAGATGATAACGTTCA   2120
DPP4              AGTACCTCCTTATTCATGGAACCGCAGATGATAACGTTCA   2120
Consensus         agtacctccttattcatggaaccgcagatgataacgttca

Isoform0010147    TTTTCAGCAGTCAGCTCAGATCACCAGAGCCTTAGTGGAT   2160
DPP4              TTTTCAGCAGTCAGCTCAGATCACCAGAGCCTTAGTGGAT   2160
Consensus         ttttcagcagtcagctcagatcaccagagccttagtggat

Isoform0010147    GCTGGCGTGGACTTCCAGGCGATGTGGTATACTGACGAAG   2200
DPP4              GCTGGCGTGGACTTCCAGGCGATGTGGTATACTGACGAAG   2200
Consensus         gctggcgtggacttccaggcgatgtggtatactgacgaag

Isoform0010147    ACCATGGAATCGCTACCAGCACAGCACACCAACATATATA   2240
DPP4              ACCATGGAATCGCTACCAGCACAGCACACCAACATATATA   2240
Consensus         accatggaatcgctaccagcacagcacaccaacatatata

Isoform0010147    CACCCACATGACCCACTTCATAAAGCAATGCTTCTCTTTA   2280
DPP4              CACCCACATGACCCACTTCATAAAGCAATGCTTCTCTTTA   2280
Consensus         cacccacatgacccacttcataaagcaatgcttctcttta

Isoform0010147    CCT..                                      2283
DPP4              CCTTA                                      2285
Consensus         cct
```

```
Isoform0010147   MKTQWKVLLGLLGAAALVTIITVPAVLLSKDDVIVDDRRT    40
DPP4             MKTQWKVLLGLLGAVALVTIITVPAVLLSKDDVIVDDRRT    40
Consensus        mktqwkvllgllga alvtiitvpavllskddvivddrrt

Isoform0010147   YTLADYLKSTIRMRNYNLRWISDHEYLYKQENNVLLFNAD    80
DPP4             YTLADYLKSTIRMRNYNLRWISDHEYLYKQENNVLLFNAD    80
Consensus        ytladylkstirmrnynlrwisdheylykqennvllfnad

Isoform0010147   HGNSSTFLENSTFDQFGHSISDYSVSPDRQFVLFEYNYVK   120
DPP4             HGNSSTFLENSTFDQFGHSISDYSVSPDRQFVLFEYNYVK   120
Consensus        hgnsstflenstfdqfghsisdysvspdrqfvlfeynyvk

Isoform0010147   KWRHSYTASYDIYDLNKRQLITEERIPNDTQLIRWSPEGH   160
DPP4             KWRHSYTASYDIYDLNKRQLITEERIPNDTQLIRWSPEGH   160
Consensus        kwrhsytasydiydlnkrqliteeripndtqlirwspegh

Isoform0010147   KLAYVWNNDIYVKNDPYSPSQRVTRDGREDAISNGITDWV   200
DPP4             KLAYVWNNDIYVKNDPYSPSQRVTRDGREDAISNGITDWV   200
Consensus        klayvwnndiyvkndpyspsqrvtrdgredaisngitdwv

Isoform0010147   YEEEIFSTHSALWWSPNGTFLAYAQFNDTDVPRIEYSVYL   240
DPP4             YEEEIFSTHSALWWSPNGTFLAYAQFNDTDVPRIEYSVYL   240
Consensus        yeeeifsthsalwwspngtflayaqfndtdvprieysvyl

Isoform0010147   DESLQYPKTIHIPYPKAGAKNPTVKLYVVNTDNLTDLEPV   280
DPP4             DESLQYPKTIHIPYPKAGAKNPTVKLYVVNTDNLTDLEPV   280
Consensus        deslqypktihipypkagaknptvklyvvntdnltdlepv

Isoform0010147   QIVAPASVLIGDHYLCDVTWATKERISLQWLRRIQNYSII   320
DPP4             QIVAPASVLIGDHYLCDVTWATKERISLQWLRRIQNYSII   320
Consensus        qivapasvligdhylcdvtwatkerislqwlrriqnysii

Isoform0010147   DICDYNESTPKWNCLVSRQHIETSATGWVGRFKPAEPHFT   360
DPP4             DICDYNESTPKWNCLVSRQHIETSATGWVGRFKPAEPHFT   360
Consensus        dicdynestpkwnclvsrqhietsatgwvgrfkpaephft

Isoform0010147   SDGNSFYKIMSNTEGYKHICLFQIDKPNCTFITKGAWEVI   400
DPP4             SDGNSFYKIMSNTEGYKHICLFQIDKPNCTFITKGAWEVI   400
Consensus        sdgnsfykimsntegykhiclfqidkpnctfitkgawevi

Isoform0010147   GIEALTNDYLYYISNEYKGMPGGRNLYKIQRNNYANVTCL   440
DPP4             GIEALTNDYLYYISNEYKGMPGGRNLYKIQRNNYANVTCL   440
Consensus        giealtndylyyisneykgmpggrnlykiqrnnyanvtcl

Isoform0010147   SCELDPERCQYYSASFSKGAKYYQLRCSGPQIPRYSLHSS   480
DPP4             SCELDPERCQYYSASFSKGAKYYQLRCSGPQIPRYSLHSS   480
Consensus        sceldpercqyysasfskgakyyqlrcsgpqiprysl hss

Isoform0010147   SNDKELRLLENNTALYETLQNIQMPRKTLDFIHLNGTKFW   520
DPP4             SNDKELRLLENNTALYETLQNIQMPRKTLDFIHLNGTKFW   520
Consensus        sndkelrllenntalyetlqniqmprktldfihlngtkfw

Isoform0010147   YQMILPPHFDKSKKYPLLIDVYAGPCSQKADATFKLSWAT   560
DPP4             YQMILPPHFDKSKKYPLLIDVYAGPCSQKADATFKLSWAT   560
Consensus        yqmilpphfdkskkypllidvyagpcsqkadatfklswat

Isoform0010147   YLASTENIIVASFDGRGSGYQGDKIMHAINRRLGTFEVED   600
DPP4             YLASTENIIVASFDGRGSGYQGDKIMHAINRRLGTFEVED   600
Consensus        ylasteniivasfdgrgsgyqgdkimhainrrlgtfeved

Isoform0010147   QIEAAKQFSKMGFVDDKRIAIWGWSYGGYVTSMVLGAGSR   640
DPP4             QIEAAKQFSKMGFVDDKRIAIWGWSYGGYVTSMVLGAGSR   640
Consensus        qieaakqfskmgfvddkriaiwgwsyggyvtsmvlgagsr

Isoform0010147   VFKCGIAVAPVSAWEFYDSVYTERYMGLPTPEDNLDHYKN   680
DPP4             VFKCGIAVAPVSAWEFYDSVYTERYMGLPTPEDNLDHYKN   680
Consensus        vfkcgiavapvsawefydsvyterymglptpednldhykn

Isoform0010147   STVMSRAENFKLVEYLLIHGTADDNVHFQQSAQITRALVD   720
DPP4             STVMSRAENFKLVEYLLIHGTADDNVHFQQSAQITRALVD   720
Consensus        stvmsraenfklveyllihgtaddnvhfqqsaqitralvd

Isoform0010147   AGVDFQAMWYTDEDHGIATSTAHQHIYTHMTHFIKQCFSL   760
DPP4             AGVDFQAMWYTDEDHGIATSTAHQHIYTHMTHFIKQCFSL   760
Consensus        agvdfqamwytdedhgiatstahqhiythmthfikqcfsl
```

Figure S5. Several published genes were compared with full-length transcripts, the comparative result of the *DPP4* gene.

```
Isoform0020210    GCAACTTCCACCATGTCCACCTCAGCGAGTTCCCACTTGA     40
Glul              ............ATGGCCACCTCAGCGAGTTCCCACTTGA     28
Consensus                     atg ccacctcagcgagttcccacttga

Isoform0020210    ACAAAGGCATCAAGCAGATGTACATGTCCCTGCCTCAGGG     80
Glul              ACAAAGGCATCAAGCAGATGTACATGTCCCTGCCTCAGGG     68
Consensus         acaaaggcatcaagcagatgtacatgtccctgcctcaggg

Isoform0020210    TGATAAAGTCCAAGCTATGTACATCTGGATTGACGGTACT    120
Glul              TGATAAAGTCCAAGCTATGTACATCTGGATTGACGGTACT    108
Consensus         tgataaagtccaagctatgtacatctggattgacggtact

Isoform0020210    GGAGAAGGACTGCGATGCAAGACCCGGACCCTGGACAGTG    160
Glul              GGAGAAGGACTGCGATGCAAGACCCGGACCCTGGACAGTG    148
Consensus         ggagaaggactgcgatgcaagacccggaccctggacagtg

Isoform0020210    AGCCCAAGAGTATAGAAGAGTTGCCCGAGTGGAATTTTGA    200
Glul              AGCCCAAGAGTATAGAAGAGTTGCCCGAGTGGAATTTTGA    188
Consensus         agcccaagagtatagaagagttgcccgagtggaattttga

Isoform0020210    TGGCTCTAGTACTATGCAGTCTGAAGGCTCCAACAGTGAC    240
Glul              TGGCTCTAGTACTATGCAGTCTGAAGGCTCCAACAGTGAC    228
Consensus         tggctctagtactatgcagtctgaaggctccaacagtgac

Isoform0020210    ATGTATCTTGTTCCTGCTACCATGTTTCGGGACCCTTTCC    280
Glul              ATGTATCTTGTTCCTGCTACCATGTTTCGGGACCCTTTCC    268
Consensus         atgtatcttgttcctgctaccatgtttcgggaccctttcc

Isoform0020210    GCAAGGACCCCAACAAGCTGGTGTTCTGTGAAGTCTTAAA    320
Glul              GCAAGGACCCCAACAAGCTGGTGTTCTGTGAAGTCTTAAA    308
Consensus         gcaaggaccccaacaagctggtgttctgtgaagtcttaaa

Isoform0020210    ATACAACCGCAAACCTGCAGAGACCAATTTAAGATATACC    360
Glul              ATACAACCGCAAACCTGCAGAGACCAATTTAAGATATACC    348
Consensus         atacaaccgcaaacctgcagagaccaatttaagatatacc

Isoform0020210    TGCAAACGGATAATGGACATGGTGAGCAATCAGCATCCCT    400
Glul              TGCAAACGGATAATGGACATGGTGAGCAATCAGCATCCCT    388
Consensus         tgcaaacggataatggacatggtgagcaatcagcatccct

Isoform0020210    GGTTTGGAATGGAGCAGGAATATACTCTCATGGGCACAGA    440
Glul              GGTTTGGAATGGAGCAGGAATATACTCTCATGGGCACAGA    428
Consensus         ggtttggaatggagcaggaatatactctcatgggcacaga

Isoform0020210    TGGGCACCCTTTTGGCTGGCCTTCCAATGGCTTCCCTGGG    480
Glul              TGGGCACCCTTTTGGCTGGCCTTCCAATGGCTTCCCTGGG    468
Consensus         tgggcaccctttggctggccttccaatggcttccctggg

Isoform0020210    CCCCAAGGTCCATACTACTGCGGTGTGGGAGCAGACAGAG    520
Glul              CCCCAAGGTCCATACTACTGCGGTGTGGGAGCAGACAGAG    508
Consensus         ccccaaggtccatactactgcggtgtgggagcagacagag

Isoform0020210    CCTACGGCAGGGATATTGTGGAGGCTCACTACCGGGCCTG    560
Glul              CCTACGGCAGGGATATTGTGGAGGCTCACTACCGGGCCTG    548
Consensus         cctacggcagggatattgtggaggctcactaccgggcctg

Isoform0020210    CTTGTACGCTGGCATCAAGATCGCAGGGACAAATGCCGAG    600
Glul              CTTGTACGCTGGCATCAAGATCGCAGGGACAAATGCCGAG    588
Consensus         cttgtacgctggcatcaagatcgcagggacaaatgccgag

Isoform0020210    GTCATGCCCGCCCAGTGGGAATTCCAAATAGGACCCTGTG    640
Glul              GTCATGCCCGCCCAGTGGGAATTCCAAATAGGACCCTGTG    628
Consensus         gtcatgcccgcccagtgggaattccaaataggaccctgtg

Isoform0020210    AAGGCATCGACATGGGAGATCATCTCTGGGTGGCCCGTTT    680
Glul              AAGGCATCGACATGGGAGATCATCTCTGGGTGGCCCGTTT    668
Consensus         aaggcatcgacatgggagatcatctctgggtggcccgttt

Isoform0020210    CATCTTGCACCGAGTGTGTGAAGACTTCGGAGTGATCGCT    720
Glul              CATCTTGCACCGAGTGTGTGAAGACTTCGGAGTGATCGCT    708
Consensus         catcttgcaccgagtgtgtgaagacttcggagtgatcgct

Isoform0020210    ACCTTTGATCCCAAGCCCATCCCTGGCAACTGGAATGGTG    760
Glul              ACCTTTGATCCCAAGCCCATCCCTGGCAACTGGAATGGTG    748
Consensus         acctttgatcccaagcccatccctggcaactggaatggtg

Isoform0020210    CAGGCTGCCACACCAACTTCAGCACCAAGGCCATGCGAGA    800
Glul              CAGGCTGCCACACCAACTTCAGCACCAAGGCCATGCGAGA    788
Consensus         caggctgccacaccaacttcagcaccaaggccatgcgaga

Isoform0020210    GGAGAACGGTCTGAAGTACATCGAGGAGTCCATTGAGAGG    840
Glul              GGAGAACGGTCTGAAGTACATCGAGGAGTCCATTGAGAGG    828
Consensus         ggagaacggtctgaagtacatcgaggagtccattgagagg

Isoform0020210    CTGAGCAAGCGGCACCAGTACCACATCCGAGCCTACGACC    880
Glul              CTGAGCAAGCGGCACCAGTACCACATCCGAGCCTACGACC    868
Consensus         ctgagcaagcggcaccagtaccacatccgagcctacgacc

Isoform0020210    CCAAGGGGGGCCGGGACAATGCCCGGCGCCTCACCGGATT    920
Glul              CCAAGGGGGGCCGGGACAATGCCCGGCGCCTCACCGGATT    908
Consensus         ccaaggggggccgggacaatgcccggcgcctcaccggatt

Isoform0020210    TAACGAAACCTCCAACATCAACGACTTCTCCGCCGGCGTG    960
Glul              TAACGAAACCTCCAACATCAACGACTTCTCCGCCGGCGTG    948
Consensus         taacgaaacctccaacatcaacgacttctccgccggcgtg

Isoform0020210    GCCAACCGTAGCGCCAGCATCCGCATTCCCCGGTCTGTGG   1000
Glul              GCCAACCGTAGCGCCAGCATCCGCATTCCCCGGTCTGTGG    988
Consensus         gccaaccgtagcgccagcatccgcattccccggtctgtgg

Isoform0020210    GCCAGGAGAAGAAGGGGTACTTTGAAGACCGGCGCCCCTC   1040
Glul              GCCAGGAGAAGAAGGGGTACTTTGAAGACCGGCGCCCCTC   1028
Consensus         gccaggagaagaaggggtactttgaagaccggcgcccctc

Isoform0020210    TGCCAACTGCGACCCCTTCGCAGTGACAGAAGCCCTCATC   1080
Glul              TGCCAACTGCGACCCCTTCGCAGTGACAGAAGCCCTCATC   1068
Consensus         tgccaactgcgaccccttcgcagtgacagaagccctcatc

Isoform0020210    CGCACGTGTCTTCTCAATGAAACTGGCGATGAGCCCTTCC   1120
Glul              CGCACGTGTCTTCTCAATGAAACTGGCGATGAGCCCTTCC   1108
Consensus         cgcacgtgtcttctcaatgaaactggcgatgagcccttcc

Isoform0020210    AGTACAAAAAC..                              1131
Glul              AGTACAAAAACTA                              1121
Consensus         agtacaaaaac
```

```
Isoform0020210   ATSTMSTSASSHLNKGIKQMYMSLPQGDKVQAMYIWIDGT   40
Glul             ....MATSASSHLNKGIKQMYMSLPQGDKVQAMYIWIDGT   36
Consensus            m tsasshlnkgikqmymslpqgdkvqamyiwidgt

Isoform0020210   GEGLRCKTRTLDSEPKSIEELPEWNFDGSSTMQSEGSNSD   80
Glul             GEGLRCKTRTLDSEPKSIEELPEWNFDGSSTMQSEGSNSD   76
Consensus        geglrcktrtldsepksieelpewnfdgsstmqsegsnsd

Isoform0020210   MYLVPATMFRDPFRKDPNKLVFCEVLKYNRKPAETNLRYT  120
Glul             MYLVPATMFRDPFRKDPNKLVFCEVLKYNRKPAETNLRYT  116
Consensus        mylvpatmfrdpfrkdpnklvfcevlkynrkpaetnlryt

Isoform0020210   CKRIMDMVSNQHPWFGMEQEYTLMGTDGHPFGWPSNGFPG  160
Glul             CKRIMDMVSNQHPWFGMEQEYTLMGTDGHPFGWPSNGFPG  156
Consensus        ckrimdmvsnqhpwfgmeqeytlmgtdghpfgwpsngfpg

Isoform0020210   PQGPYYCGVGADRAYGRDIVEAHYRACLYAGIKIAGTNAE  200
Glul             PQGPYYCGVGADRAYGRDIVEAHYRACLYAGIKIAGTNAE  196
Consensus        pqgpyycgvgadraygrdiveahyraclyagikiagtnae

Isoform0020210   VMPAQWEFQIGPCEGIDMGDHLWVARFILHRVCEDFGVIA  240
Glul             VMPAQWEFQIGPCEGIDMGDHLWVARFILHRVCEDFGVIA  236
Consensus        vmpaqwefqigpcegidmgdhlwvarfilhrvcedfgvia

Isoform0020210   TFDPKPIPGNWNGAGCHTNFSTKAMREENGLKYIEESIER  280
Glul             TFDPKPIPGNWNGAGCHTNFSTKAMREENGLKYIEESIER  276
Consensus        tfdpkpipgnwngagchtnfstkamreenglkyieesier

Isoform0020210   LSKRHQYHIRAYDPKGGRDNARRLTGFNETSNINDFSAGV  320
Glul             LSKRHQYHIRAYDPKGGRDNARRLTGFNETSNINDFSAGV  316
Consensus        lskrhqyhiraydpkggrdnarrltgfnetsnindfsagv

Isoform0020210   ANRSASIRIPRSVGQEKKGYFEDRRPSANCDPFAVTEALI  360
Glul             ANRSASIRIPRSVGQEKKGYFEDRRPSANCDPFAVTEALI  356
Consensus        anrsasiriprsvgqekkgyfedrrpsancdpfavteali

Isoform0020210   RTCLLNETGDEPFQYK                          376
Glul             RTCLLNETGDEPFQYK                          372
Consensus        rtcllnetgdepfqyk
```

Figure S6. Several published genes were compared with full-length transcripts, the comparative result of the *Glul* gene.

```
Isoform0025243   ATGTATTTCATTAATCTACTAACAATAATCGTTCCCATCC    40
ND1              ATGTATTTCATTAATCTACTAACAATAATCGTTCCCATCC    40
Consensus        atgtatttcattaatctactaacaataatcgttcccatcc

Isoform0025243   TACTAGCCGTAGCATTTTTAACCCTGCTAGAACGAAAAGT    80
ND1              TACTAGCCGTAGCATTTTTAACCCTGCTAGAACGAAAAGT    80
Consensus        tactagccgtagcatttttaaccctgctagaacgaaaagt

Isoform0025243   ACTAGGTTATATACAACTTCGAAAAGGACCAAACATTGTT   120
ND1              ACTAGGTTATATACAACTTCGAAAAGGACCAAACATTGTT   120
Consensus        actaggttatatacaacttcgaaaaggaccaaacattgtt

Isoform0025243   GGTCCTTACGGTTTACTGCAGCCAATCGCTGACGCAGTCA   160
ND1              GGTCCTTACGGTTTACTGCAGCCAATCGCTGACGCAGTCA   160
Consensus        ggtccttacggtttactgcagccaatcgctgacgcagtca

Isoform0025243   AATTATTTACTAAAGAACCTATACAACCACTAACATCATC   200
ND1              AATTATTTACTAAAGAACCTATACAACCACTAACATCATC   200
Consensus        aattatttactaaagaacctatacaaccactaacatcatc

Isoform0025243   TCTTACCTTATTCATTATTGCTCCTACTTTAGCCCTGACT   240
ND1              TCTTACCTTATTCATTATTGCTCCTACTTTAGCCCTGACT   240
Consensus        tcttaccttattcattattgctcctactttagccctgact

Isoform0025243   TTAGCCCTAATAATATGAATCCCATTACCCATACCACATC   280
ND1              TTAGCCCTAATAATATGAATCCCATTACCCATACCACATC   280
Consensus        ttagccctaataatatgaatcccattacccataccacatc

Isoform0025243   CCCTAATTAACATAAACCTAAGCATACTATTTATACTAGC   320
ND1              CCCTAATTAACATAAACCTAAGCATACTATTTATACTAGC   320
Consensus        ccctaattaacataaacctaagcatactatttatactagc

Isoform0025243   TCTATCAAGTCTGGCCGTCTACGCTATTCTATGATCAGGC   360
ND1              TCTATCAAGTCTGGCCGTCTACGCTATTCTATGATCAGGC   360
Consensus        tctatcaagtctggccgtctacgctattctatgatcaggc

Isoform0025243   TGAGCTTCAAATTCAAAATACGCATTAATTGGAGCCTTAC   400
ND1              TGAGCTTCAAATTCAAAATACGCATTAATTGGAGCCTTAC   400
Consensus        tgagcttcaaattcaaaatacgcattaattggagccttac

Isoform0025243   GAGCAGTAGCCCAAACAATCTCCTATGAAGTAACCCTAGC   440
ND1              GAGCAGTAGCCCAAACAATCTCCTATGAAGTAACCCTAGC   440
Consensus        gagcagtagcccaaacaatctcctatgaagtaaccctagc

Isoform0025243   CATTATTATTCTATCCATCCTGCTTATAAATGGCTCATTC   480
ND1              CATTATTATTCTATCCATCCTGCTTATAAATGGCTCATTC   480
Consensus        cattattattctatccatcctgcttataaatggctcattc

Isoform0025243   ACATTAACCATACTAATTACAACACAAGAATATATCTGAT   520
ND1              ACATTAACCACACTAATTACAACACAAGAATATATCTGAT   520
Consensus        acattaacca actaattacaacacaagaatatatctgat

Isoform0025243   TAATTATTCCCTCATGACCTCTGGCTATAATATGATTTAT   560
ND1              TAATTATCCCCTCATGACCTCTGGCCATAATATGATTTAT   560
Consensus        taattat ccctcatgacctctggc ataatatgatttat

Isoform0025243   CTCAACTTTAGCAGAAACTAACCGAGCCCCCTTTGACTTA   600
ND1              CTCAACTTTAGCAGAAACTAACCGAGCCCCCTTTGACTTA   600
Consensus        ctcaactttagcagaaactaaccgagccccctttgactta

Isoform0025243   ACTGAGGGAGAATCAGAATTAGTATCTGGTTTCAACGTAG   640
ND1              ACTGAGGGAGAATCAGAATTAGTATCTGGTTTCAACGTAG   640
Consensus        actgagggagaatcagaattagtatctggtttcaacgtag

Isoform0025243   AATATGCAGGAGGACCCTTCGCTCTCTTCTTTCTAGCAGA   680
ND1              AATATGCAGGAGGACCCTTCGCTCTCTTCTTTCTAGCAGA   680
Consensus        aatatgcaggaggacccttcgctctcttctttctagcaga

Isoform0025243   ATATGCTAATATTATCATAATAAATGCCCTCACAATTATC   720
ND1              ATATGCTAATATTATCATAATAAATGCCCTCACAATTATC   720
Consensus        atatgctaatattatcataataaatgccctcacaattatc

Isoform0025243   CTATTCTTAGGCGCATATAATAATCCTATATTCTCAGAAC   760
ND1              CTATTCTTAGGCGCATATAATAATCCTATATTCTCAGAAC   760
Consensus        ctattcttaggcgcatataataatcctatattctcagaac

Isoform0025243   TTTACACCACCAACTTCACCACTAAAACTCTCTTATTCAC   800
ND1              TTTACACCACCAACTTCACCACTAAAACTCTCTTATTCAC   800
Consensus        tttacaccaccaacttcaccactaaaactctcttattcac

Isoform0025243   AATAATATTTTTATGAATTCGAGCATCTTACCCTCGATTC   840
ND1              AATAATATTTTTATGAATTCGAGCATCTTACCCTCGATTC   840
Consensus        aataatatttttatgaattcgagcatcttaccctcgattc

Isoform0025243   CGATATGACCAACTAATACACCTACTATGAAAAAACTTTC   880
ND1              CGATATGACCAACTAATACACCTACTATGAAAAAACTTTC   880
Consensus        cgatatgaccaactaatacacctactatgaaaaaactttc

Isoform0025243   TACCCCTTACTTTAGTAATATGTATATGACATGTAACCTT   920
ND1              TACCCCTTACTTTAGTAATATGTATATGACATGTAACTTT   920
Consensus        taccccttactttagtaatatgtatatgacatgtaac tt

Isoform0025243   ACCAATTATTCTAGCAAGTATCCCACCCATAACA..       954
ND1              ACCAATTATTCTAGCAAGTATCCCACCCATAACATA       956
Consensus        accaattattctagcaagtatcccacccataaca
```

```
Isoform0025243    MYFINLLTIIVPILLAVAFLTLLERKVLGYIQLRKGPNIV     40
ND1               MYFINLLTIIVPILLAVAFLTLLERKVLGYIQLRKGPNIV     40
Consensus         myfinlltiivpillavafltllerkvlgyiqlrkgpniv

Isoform0025243    GPYGLLQPIADAVKLFTKEPIQPLTSSLTLFIIAPTLALT     80
ND1               GPYGLLQPIADAVKLFTKEPIQPLTSSLTLFIIAPTLALT     80
Consensus         gpygllqpiadavklftkepiqpltssltlfiiaptlalt

Isoform0025243    LALIIIPLPIPHPLININLSILFILALSSLAVYAILSGAS    120
ND1               LALIIIPLPIPHPLININLSILFILALSSLAVYAILSGAS    120
Consensus         laliiiplpiphplininlsilfilalsslavyailsgas

Isoform0025243    NSKYALIGALRAVAQTISYEVTLAIIILSILLINGSFTLT    160
ND1               NSKYALIGALRAVAQTISYEVTLAIIILSILLINGSFTLT    160
Consensus         nskyaligalravaqtisyevtlaiiilsillingsftlt

Isoform0025243    ILITTQEYILIIPSPLAIIFISTLAETNRAPFDLTEGESE    200
ND1               TLITTQEYILIIPSPLAIIFISTLAETNRAPFDLTEGESE    200
Consensus          littqeyiliipsplaiifistlaetnrapfdltegese

Isoform0025243    LVSGFNVEYAGGPFALFFLAEYANIIIINALTIILFLGAY    240
ND1               LVSGFNVEYAGGPFALFFLAEYANIIIINALTIILFLGAY    240
Consensus         lvsgfnveyaggpfalfflaeyaniiiinaltiilflgay

Isoform0025243    NNPIFSELYTTNFTTKTLLFTIIFLIRASYPRFRYDQLIH    280
ND1               NNPIFSELYTTNFTTKTLLFTIIFLIRASYPRFRYDQLIH    280
Consensus         nnpifselyttnfttktllftiiflirasyprfrydqlih

Isoform0025243    LLKNFLPLTLVICIHVTLPIILASIPPI                308
ND1               LLKNFLPLTLVICIHVTLPIILASIPPI                308
Consensus         llknflpltlvicihvtlpiilasippi
```

Figure S7. Several published genes were compared with full-length transcripts, the comparative result of the *ND1* gene.

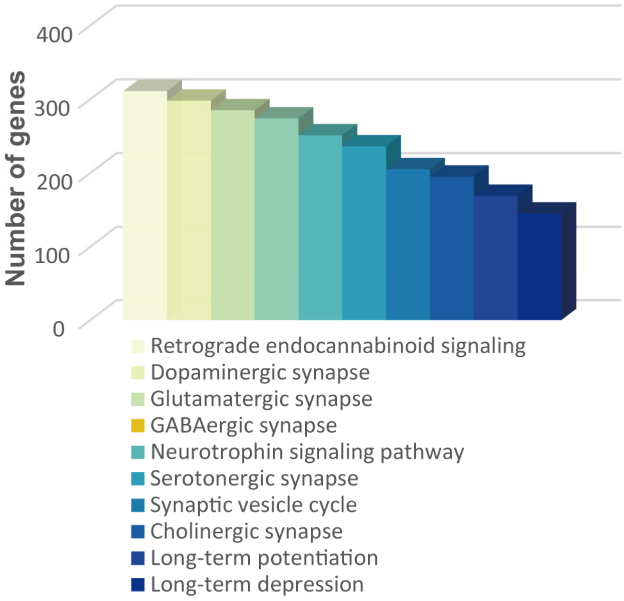

Figure S8. The proportion of full-length transcripts annotated in the metabolic pathways of the neural-related system.

```
Isoform0022807    ATGGATTGGGGTACCCTGCACACTTTCATCGGGGGTGTGA     40
GJB6              ATGGATTGGGGTACCCTGCACACTTTCATCGGGGGTGTGA     40
Consensus         atggattggggtaccctgcacactttcatcgggggtgtga

Isoform0022807    ACAAACACTCCACCAGCATCGGGAAGGTGTGGATCACCGT     80
GJB6              ACAAACACTCCACCAGCATCGGGAAGGTGTGGATCACCGT     80
Consensus         acaaacactccaccagcatcgggaaggtgtggatcaccgt

Isoform0022807    CATCTTCATCTTCCGTGTCATGATCCTCGTGGTGGCCGCT    120
GJB6              CATCTTCATCTTCCGTGTCATGATCCTCGTGGTGGCCGCT    120
Consensus         catcttcatcttccgtgtcatgatcctcgtggtggccgct

Isoform0022807    CAGGAAGTGTGGGGAGATGAACAGGAAGACTTTGTGTGCA    160
GJB6              CAGGAAGTGTGGGGAGATGAACAGGAAGACTTTGTGTGCA    160
Consensus         caggaagtgtggggagatgaacaggaagactttgtgtgca

Isoform0022807    ACACTCTGCAGCCAGGATGCAGAAACGTGTGCTATGACCA    200
GJB6              ACACTCTGCAGCCAGGATGCAGAAACGTGTGCTATGACCA    200
Consensus         acactctgcagccaggatgcagaaacgtgtgctatgacca

Isoform0022807    CTTCTTCCCCGTGTCCCACATCCGGCTGTGGGCCCTGCAG    240
GJB6              CTTCTTCCCCGTGTCCCACATCCGGCTGTGGGCCCTGCAG    240
Consensus         cttcttccccgtgtcccacatccggctgtgggccctgcag

Isoform0022807    CTCATCTTCGTCTCCACCCCTGCCCTGCTTGTGGCCATGC    280
GJB6              CTCATCTTCGTCTCCACCCCTGCCCTGCTTGTGGCCATGC    280
Consensus         ctcatcttcgtctccacccctgccctgcttgtggccatgc

Isoform0022807    ACGTTGCCTACTACCGGCAGGAGGCCGCCCGCAAATTCAG    320
GJB6              ACGTTGCCTACTACCGGCAGGAGGCCGCCCGCAAATTCAG    320
Consensus         acgttgcctactaccggcaggaggccgcccgcaaattcag

Isoform0022807    ACGAGGAGAGAAGAGAAATGAATTCAAAGACTTGGAAGAC    360
GJB6              ACGAGGAGAGAAGAGAAATGAATTCAAAGACTTGGAAGAC    360
Consensus         acgaggagagaagagaaatgaattcaaagacttggaagac

Isoform0022807    ATTAAAAAGCAGAAGGTTCGGATCGAGGGAGCCCTGTGGT    400
GJB6              ATTAAAAAGCAGAAGGTTCGGATCGAGGGAGCCCTGTGGT    400
Consensus         attaaaaagcagaaggttcggatcgagggagccctgtggt

Isoform0022807    GGACGTACACCAGCAGCATATTTTTCCGAATCATCTTCGA    440
GJB6              GGACGTACACCAGCAGCATATTTTTCCGAATCATCTTCGA    440
Consensus         ggacgtacaccagcagcatatttttccgaatcatcttcga

Isoform0022807    AGCCTCCTTCATGTATGTGTTTTACTTCCTATACAATGGG    480
GJB6              AGCCTCCTTCATGTATGTGTTTTACTTCCTATACAATGGG    480
Consensus         agcctccttcatgtatgtgttttacttcctatacaatggg

Isoform0022807    TACCACCTGCCCTGGGTGCTGAAATGTGGGGTTGATCCTT    520
GJB6              TACCACCTGCCCTGGGTGCTGAAATGTGGGGTTGATCCTT    520
Consensus         taccacctgccctgggtgctgaaatgtggggttgatcctt

Isoform0022807    GCCCCAATCTTGTTGACTGCTTCATCTCCAGACCTACGGA    560
GJB6              GCCCCAATCTTGTTGACTGCTTCATCTCCAGACCTACGGA    560
Consensus         gccccaatcttgttgactgcttcatctccagacctacgga

Isoform0022807    GAAAACCGTGTTCACCATTTTTATGATCTCTGCCTCGGTG    600
GJB6              GAAAACCGTGTTCACCATTTTTATGATCTCTGCCTCGGTG    600
Consensus         gaaaaccgtgttcaccatttttatgatctctgcctcggtg

Isoform0022807    ATTTGCATGCTACTCAATGTGGCCGAGTTGTGCTACCTGC    640
GJB6              ATTTGCATGCTACTCAATGTGGCCGAGTTGTGCTACCTGC    640
Consensus         atttgcatgctactcaatgtggccgagttgtgctacctgc

Isoform0022807    TGCTGAAAGTGTGTTTCAGGAAATCAAAAAGAGCCCAGAC    680
GJB6              TGCTGAAAGTGTGTTTCAGGAAATCAAAAAAAGCCCAGAC    680
Consensus         tgctgaaagtgtgtttcaggaaatcaaaaa agcccagac

Isoform0022807    ACAAAGAAACCACCCCAGTCATGCCCTAAAGGAGAGTAAG    720
GJB6              ACAAAGAAACCACCCCAGTCATGCCCTAAAGGAGAGTAAG    720
Consensus         acaaagaaaccaccccagtcatgccctaaaggagagtaag

Isoform0022807    CAAAATGAAATGAATGAGCTGATTTCAGATAGTGGTCAAA    760
GJB6              CAAAATGAAATGAATGAGCTGATTTCAGATAGTGGTCAAA    760
Consensus         caaaatgaaatgaatgagctgatttcagatagtggtcaaa

Isoform0022807    ATGCAACCACTGGTTTTCCAAGT..                   783
GJB6              ATGCAACCACTGGTTTTCCAAGTTA                   785
Consensus         atgcaaccactggttttccaagt
```

```
Isoform0022807    MDWGTLHTFIGGVNKHSTSIGKVWITVIFIFRVMILVVAA     40
GJB6              MDWGTLHTFIGGVNKHSTSIGKVWITVIFIFRVMILVVAA     40
Consensus         mdwgtlhtfiggvnkhstsigkvwitvififrvmilvvaa

Isoform0022807    QEVWGDEQEDFVCNTLQPGCRNVCYDHFFPVSHIRLWALQ     80
GJB6              QEVWGDEQEDFVCNTLQPGCRNVCYDHFFPVSHIRLWALQ     80
Consensus         qevwgdeqedfvcntlqpgcrnvcydhffpvshirlwalq

Isoform0022807    LIFVSTPALLVAMHVAYYRQEAARKFRRGEKRNEFKDLED    120
GJB6              LIFVSTPALLVAMHVAYYRQEAARKFRRGEKRNEFKDLED    120
Consensus         lifvstpallvamhvayyrqeaarkfrrgekrnefkdled

Isoform0022807    IKKQKVRIEGALWWTYTSSIFFRIIFEASFMYVFYFLYNG    160
GJB6              IKKQKVRIEGALWWTYTSSIFFRIIFEASFMYVFYFLYNG    160
Consensus         ikkqkvriegalwwtytssiffriifeasfmyvfyflyng

Isoform0022807    YHLPWVLKCGVDPCPNLVDCFISRPTEKTVFTIFMISASV    200
GJB6              YHLPWVLKCGVDPCPNLVDCFISRPTEKTVFTIFMISASV    200
Consensus         yhlpwvlkcgvdpcpnlvdcfisrptektvftifmisasv

Isoform0022807    ICMLLNVAELCYLLLKVCFRKSKRAQTQRNHPSHALKESK    240
GJB6              ICMLLNVAELCYLLLKVCFRKSKKAQTQRNHPSHALKESK    240
Consensus         icmllnvaelcylllkvcfrksk aqtqrnhpshalkesk

Isoform0022807    QNEMNELISDSGQNATTGFP                        260
GJB6              QNEMNELISDSGQNATTGFP                        260
Consensus         qnemnelisdsgqnattgfp
```

Figure S9. Several published genes were compared with full-length transcripts, the comparative result of the *GJB6* gene.

```
Isoform0013754  ATAACGCATTGCGCAGGGTGCACCACCGCCTGGTCTCCCT    40
SK2             ATAACGCATTGCGCAGGGTGCACCACCGCCTGGTCTCCCT    40
Consensus       ataacgcattgcgcagggtgcaccaccgcctggtctccct

Isoform0013754  GCAGCTTTAATAGCCCTGACATGGAAACCCCATTGCAGTT    80
SK2             GCAGCTTTAATAGCCCTGACATGGAAACCCCATTGCAGTT    80
Consensus       gcagctttaatagccctgacatggaaaccccattgcagtt

Isoform0013754  TCAGCGCGGCTTCTTCTCAGAGCAGCCGCCGCCGCCGCGC   120
SK2             TCAGCGCGGCTTCTTCTCAGAGC............CGCGC   108
Consensus       tcagcgcggcttcttctcagagc            cgcgc

Isoform0013754  TCCTCACACCTGCATTGCCAGCAGCAGCAGCAGAGCCAGG   160
SK2             TCCTCACACCTGCATTGCCAGCAGCAACAG...AGCCAGG   145
Consensus       tcctcacacctgcattgccagcagca cag   agccagg

Isoform0013754  ACAAGCCGTGCCCGCCCTTCGCGTCCCTCCCGCACGCACA   200
SK2             ACAAGCCGTGCCCGCCCTTCGCGTCCCTCCCGCACGCACA   185
Consensus       acaagccgtgcccgcccttcgcgtccctcccgcacgcaca

Isoform0013754  CCACCACCCGCACCTCGCGCACCAGCAGCCGGGTAGCGGC   240
SK2             CCACCACCCGCACCTCGCGCACCAGCAGCCGGGTAGCGGC   225
Consensus       ccaccacccgcacctcgcgcaccagcagccgggtagcggc

Isoform0013754  GGCAGCAGCCCATGCCTCCGGTGCAACAGCTGCGCCTCCT   280
SK2             GGCAGCAGCCCATGCCTCCGGTGCAACAGCTGCGCCTCCT   265
Consensus       ggcagcagcccatgcctccggtgcaacagctgcgcctcct

Isoform0013754  CCGGTGCCCCCGGGGCGGGGGCGGGGGCGGGGGATAACCT   320
SK2             CCGGTGCCCCCG.........CGGGGGCGGGGGATAACCT   296
Consensus       ccggtgcccccg         cgggggcgggggataacct

Isoform0013754  GTCCCTTCTGCTCCGCACCTCCTCGCCCGGCGGCGCCTTC   360
SK2             GTCCCTTCTGCTCCGCACCTCCTCGCCCGGCGGC...TTC   333
Consensus       gtcccttctgctccgcacctcctcgcccggcggc   ttc

Isoform0013754  CGGACCCGCACCTCCTCGCCGCTGTCGGGCTCGTCGTGCT   400
SK2             CGGACCCGCACCTCCTCGCCGCTGTCGGGCTCGTCGTGCT   373
Consensus       cggacccgcacctcctcgccgctgtcgggctcgtcgtgct

Isoform0013754  GCTGCTGCTCGTCGCGCCGGGGCAGCCAGCTCAATGTGAG   440
SK2             GCTGCTGCT...CGCGCCGGGGCAGCCAGCTCAATGTGAG   410
Consensus       gctgctgct   cgcgccggggcagccagctcaatgtgag

Isoform0013754  CGAGCTGACTCCGTCCAGCCATGCCAGTGCGCTCCGGCAG   480
SK2             CGAGCTGACTCCGTCCAGCCATGCCAGTGCGCTCCGGCAG   450
Consensus       cgagctgactccgtccagccatgccagtgcgctccggcag

Isoform0013754  CAGTACGCGCAGCAGCCCGCGTCCGCCTCCCTGTACCACC   520
SK2             CAGTACGCGCAGCAG...GCGTCCGCCTCCCTGTACCACC   487
Consensus       cagtacgcgcagcag   gcgtccgcctccctgtaccacc

Isoform0013754  AGTGCCACAGCCTGCAGCCCGCCGCCAGCCCCACGGGCAG   560
SK2             AGTGCCACAGCCTGCAGCCCGCCGCCAGCCCCACGGGCAG   527
Consensus       agtgccacagcctgcagcccgccgccagccccacgggcag

Isoform0013754  CCTCGGCAGCCTGGGCTCCGGGCCCCCGCTCTCGCACCAC   600
SK2             CCTCGGCAGCCTGGGCTCCGGGCCCCCGCTCTCGCACCAC   567
Consensus       cctcggcagcctgggctccgggcccccgctctcgcaccac

Isoform0013754  CACCACCACCCCCACTTGGCGCACCACCAGCACCACCAGT   640
SK2             CAC............TTGGCGCACCACCAGCACCACCAGT   595
Consensus       cac            ttggcgcaccaccagcaccaccagt

Isoform0013754  CCCAGGCACGCCGCGAGAGCAACCCCTTCACCGAAATAGC   680
SK2             CCCAGGCACGCCGCGAGAGCAACCCCTTCACCGAAATAGC   635
Consensus       cccaggcacgccgcgagagcaaccccttcaccgaaatagc

Isoform0013754  CATGAGCAGCTGCAGGTACAACGGGGGCGTCATGCGTCCG   720
SK2             CATGAGCAGCTGCAGGTACAACGGGGGCGTCATGCGTCCG   675
Consensus       catgagcagctgcaggtacaacgggggcgtcatgcgtccg

Isoform0013754  CTGAGCAACTTGAGCGCGTCCCGCCGAAACCTGCACGAGA   760
SK2             CTGAGCAACTTGAGCGCGTCCCGCCGAAACCTGCACGAGA   715
Consensus       ctgagcaacttgagcgcgtcccgccgaaacctgcacgaga

Isoform0013754  TGGACTCGGAGGCACAGCCCCTCCAGCCACCCGCGTCCGT   800
SK2             TGGACTCGGAGGCACAGCCCCTCCAGCCACCCGCGTCCGT   755
Consensus       tggactcggaggcacagcccctccagccacccgcgtccgt

Isoform0013754  CGGAGGAGGCGGCGGCGCGTCCTCTCCGTCTGCGGTCGCC   840
SK2             CGGAGG...TGGCGGCGCGTCCTCTCCGTCTGCA......   786
Consensus       cggagg    ggcggcgcgtcctctccgtctgc

Isoform0013754  GCTGCAGCTTCGTCCTCAGCCCCCGAGATCGTGGTGTCAA   880
SK2             .........TCGTCCTCAGCCCCCGAGATCGTGGTGTCAA   817
Consensus                tcgtcctcagcccccgagatcgtggtgtcaa

Isoform0013754  AGCCGGAGCACAACAACTCCAATAACCTGGCGCTCTATGG   920
SK2             AGCCGGAGCACAACAACTCCAATAACCTGGCGCTCTATGG   857
Consensus       agccggagcacaacaactccaataacctggcgctctatgg

Isoform0013754  AACCGGCGGCGGAGGCAGCACTGGAGGCGGCGGCGGCAGC   960
SK2             AACCGGCGGCGGAG..................GCGGCGGC   879
Consensus       aaccggcggcggag                  gcggc gc

Isoform0013754  GGCAGCGGGCATGGCAGCAGCAGTGGCACCAAGTCTAGCA  1000
SK2             GGCAGCGGGCATGGCAGCAGCAGTGGCACCAAGTCTAGCA   919
Consensus       ggcagcgggcatggcagcagcagtggcaccaagtctagca

Isoform0013754  AGAAGAAGAACCAGAACATCGGCTACAAGCTGGGCCACCG  1040
SK2             AGAAGAAGAACCAGAACATCGGCTACAAGCTGGGCCACCG   959
Consensus       agaagaagaaccagaacatcggctacaagctgggccaccg

Isoform0013754  GCGTGCCCTTTTCGAGAAGCGCAAGCGTCTCAGTGACTAC  1080
SK2             GCGTGCCCTCTTCGAGAAGCGCAAGCGTCTCAGTGACTAC   999
Consensus       gcgtgccct ttcgagaagcgcaagcgtctcagtgactac

Isoform0013754  GCGCTCATCTTCGGCATGTTCGGCATCGTGGTTATGGTCA  1120
SK2             GCGCTCATCTTCGGCATGTTCGGCATCGTGGTTATGGTCA  1039
Consensus       gcgctcatcttcggcatgttcggcatcgtggttatggtca

Isoform0013754  TTGAGACCGAGCTGTCGTGGGGCGCCTACGACAAGGCGTC  1160
SK2             TTGAGACCGAGCTGTCGTGGGGCGCCTACGACAAGGCGTC  1079
Consensus       ttgagaccgagctgtcgtggggcgcctacgacaaggcgtc

Isoform0013754  GCTGTATTCCTTAGCTCTGAAATGCCTTATCAGTCTCTCC  1200
SK2             GCTGTATTCCTTAGCTCTGAAATGCCTTATCAGTCTCTCC  1119
Consensus       gctgtattccttagctctgaaatgccttatcagtctctcc

Isoform0013754  ACGATCATTCTGCTCGGTCTGATCATCGTGTACCACGCCA  1240
SK2             ACGATCATTCTGCTCGGTCTGATCATCGTGTACCACGCCA  1159
Consensus       acgatcattctgctcggtctgatcatcgtgtaccacgcca

Isoform0013754  GGGAAATACAGTTGTTCATGGTGGACAATGGAGCAGATGA  1280
SK2             GGGAAATACAGTTGTTCATGGTGGACAATGGAGCAGATGA  1199
Consensus       gggaaatacagttgttcatggtggacaatggagcagatga

Isoform0013754  CTGGAGAATAGCCATGACTTATGAGCGTATTTTCTTCATC  1320
SK2             CTGGAGAATAGCCATGACTTATGAGCGTATTTTCTTCATC  1239
Consensus       ctggagaatagccatgacttatgagcgtattttcttcatc

Isoform0013754  TGCTTGGAAATACTGGTGTGTGCTATTCATCCCATACCTG  1360
SK2             TGCTTGGAAATACTGGTGTGTGCTATTCATCCCATACCTG  1279
Consensus       tgcttggaaatactggtgtgtgctattcatcccatacctg

Isoform0013754  GGAATTATACGTTCACATGGACGGCCCGGCTTGCCTTCTC  1400
SK2             GGAATTATACGTTCACATGGACGGCCCGGCTTGCCTTCTC  1319
Consensus       ggaattatacgttcacatggacggcccggcttgccttctc

Isoform0013754  CTATGCCCCATCCACAACCACCGCTGATGTGGATATTATT  1440
SK2             CTATGCCCCATCCACAACCACCGCCGATGTGGATATTATT  1359
Consensus       ctatgccccatccacaaccaccgc gatgtggatattatt

Isoform0013754  TTATCTATACCAATGTTCTTAAGACTCTATCTGATTGCCA  1480
SK2             TTATCTATACCAATGTTCTTAAGACTCTATCTGATTGCCA  1399
Consensus       ttatctataccaatgttcttaagactctatctgattgcca

Isoform0013754  GAGTCATGCTTTTACATAGCAAACTTTTCACTGATGCCTC  1520
SK2             GAGTCATGCTTTTACATAGCAAACTTTTCACTGATGCCTC  1439
Consensus       gagtcatgcttttacatagcaaacttttcactgatgcctc

Isoform0013754  CTCTAGAAGCATTGGAGCACTTAATAAGATAAACTTCAAT  1560
SK2             CTCTAGAAGCATTGGAGCACTTAATAAGATAAACTTCAAT  1479
Consensus       ctctagaagcattggagcacttaataagataaacttcaat

Isoform0013754  ACGCGTTTTGTTATGAAGACTTTAATGACTATATGCCCAG  1600
SK2             ACGCGTTTTGTTATGAAGACTTTAATGACTATATGCCCAG  1519
Consensus       acgcgttttgttatgaagactttaatgactatatgcccag

Isoform0013754  GAACTGTACTCTTGGTTTTTAGTATCTCATTATGGATAAT  1640
SK2             GAACTGTACTCTTGGTTTTTAGTATCTCATTATGGATAAT  1559
Consensus       gaactgtactcttggtttttagtatctcattatggataat

Isoform0013754  TGCCGCATGGACTGTCCGAGCTTGTGAAAGGTACCATGAT  1680
SK2             TGCCGCATGGACTGTCCGAGCTTGTGAAAGGTACCATGAT  1599
Consensus       tgccgcatggactgtccgagcttgtgaaaggtaccatgat

Isoform0013754  CAACAGGATGTTACTAGCAACTTCCTTGGAGCAATGTGGT  1720
SK2             CAACAGGATGTTACTAGCAACTTCCTTGGAGCAATGTGGT  1639
Consensus       caacaggatgttactagcaacttccttggagcaatgtggt

Isoform0013754  TGATATCAATAACTTTTCTCTCCATTGGTTATGGTGACAT  1760
SK2             TGATATCAATAACTTTTCTCTCCATTGGTTATGGTGACAT  1679
Consensus       tgatatcaataacttttctctccattggttatggtgacat

Isoform0013754  GGTACCTAACACATACTGTGGGAAAGGAGTCTGTTTGCTT  1800
SK2             GGTACCTAACACATACTGTGGGAAAGGAGTCTGTTTGCTT  1719
Consensus       ggtacctaacacatactgtgggaaaggagtctgtttgctt

Isoform0013754  ACTGGAATTATGGGTGCTGGTTGCACAGCCCTGGTGGTAG  1840
SK2             ACTGGAATTATGGGTGCTGGTTGCACAGCCCTGGTGGTAG  1759
Consensus       actggaattatgggtgctggttgcacagccctggtggtag

Isoform0013754  CTGTAGTGGCAAGGAAGCTAGAACTTACCAAAGCAGAAAA  1880
SK2             CTGTAGTGGCAAGGAAGCTAGAACTTACCAAAGCAGAAAA  1799
Consensus       ctgtagtggcaaggaagctagaacttaccaaagcagaaaa

Isoform0013754  GCACGTGCACAACTTCATGATGGATACTCAGCTGACAAAA  1920
SK2             GCACGTGCACAACTTCATGATGGATACTCAGCTGACAAAA  1839
Consensus       gcacgtgcacaacttcatgatggatactcagctgacaaaa

Isoform0013754  AGAGTGAAAAACGCAGCTGCCAATGTACTCAGGGAAACAT  1960
SK2             AGAGTGAAAAACGCAGCTGCCAATGTACTCAGGGAAACAT  1879
Consensus       agagtgaaaaacgcagctgccaatgtactcagggaaacat

Isoform0013754  GGCTAATTTACAAAAATACAAAGCTAGTAAAAAAGATAGA  2000
SK2             GGCTAATTTACAAAAATACAAAGCTAGTAAAAAAGATAGA  1919
Consensus       ggctaatttacaaaaatacaaagctagtaaaaaagataga

Isoform0013754  TCATGCAAAAGTAAGAAAACATCAACGAAAATTCTTGCAA  2040
SK2             TCATGCAAAAGTAAGAAAACATCAACGAAAATTCTTGCAA  1959
Consensus       tcatgcaaaagtaagaaaacatcaacgaaaattcttgcaa

Isoform0013754  GCTATTCATCAATTAAGAAGTGTAAAAATGGAGCAGAGGA  2080
SK2             GCTATTCATCAATTAAGAAGTGTAAAAATGGAGCAGAGGA  1999
Consensus       gctattcatcaattaagaagtgtaaaaatggagcagagga

Isoform0013754  AACTGAATGACCAAGCAAATACCTTGGTGGACCTGGCAAA  2120
SK2             AACTGAATGACCAAGCAAATACCTTGGTGGACCTGGCAAA  2039
Consensus       aactgaatgaccaagcaaataccttggtggacctggcaaa

Isoform0013754  GACCCAGAATATCATGTATGACATGATTTCCGACTTAAAC  2160
SK2             GACCCAGAATATCATGTATGACATGATTTCCGACTTAAAC  2079
Consensus       gacccagaatatcatgtatgacatgatttccgacttaaac

Isoform0013754  GAAAGGAGTGAAGACTTCGAGAAGAGGATTGTTACCCTGG  2200
SK2             GAAAGGAGTGAAGACTTCGAGAAGAGGATTGTTACCCTGG  2119
Consensus       gaaaggagtgaagacttcgagaagaggattgttaccctgg

Isoform0013754  AAACGAAATTAGAGACTTTGATTGGTAGCATCCACGCCCT  2240
SK2             AAACGAAATTAGAGACTTTGATTGGTAGCATCCACGCCCT  2159
Consensus       aaacgaaattagagactttgattggtagcatccacgccct

Isoform0013754  CCCCGGGCTCATCAGCCAGACCATCAGACAGCAGCAGAGA  2280
SK2             CCCTGGGCTCATCAGCCAGACCATCAGACAGCAGCAGAGA  2199
Consensus       ccc gggctcatcagccagaccatcagacagcagcagaga

Isoform0013754  GACTTCATTGAGGCTCAGATGGAGAACTACGACAAGCATG  2320
SK2             GATTTCATTGAGGCTCAGATGGAGAACTACGACAAGCATG  2239
Consensus       ga ttcattgaggctcagatggagaactacgacaagcatg

Isoform0013754  TCGCCTACAATGCCGAGCGGTCCCGG                2346
SK2             TCGCCTACAATGCCGAGCGGTCCCGG                2265
Consensus       tcgcctacaatgccgagcggtcccgg
```

```
Isoform0013754    ITHCAGCTTAWSPCSFNSPDMETPLQFQRGFFSEQPPPPR    40
SK2               ITHCAGCTTAWSPCSFNSPDMETPLQFQRGFFSE....PR    36
Consensus         ithcagcttawspcsfnspdmetplqfqrgffse    pr

Isoform0013754    SSHLHCQQQQQSQDKPCPPFASLPHAHHHPHLAHQQPGSG    80
SK2               SSHLHCQQQQ.SQDKPCPPFASLPHAHHHPHLAHQQPGSG    75
Consensus         sshlhcqqqq sqdkpcppfaslphahhhphlahqqpgsg

Isoform0013754    GSSPCLRCNSCASSGAPGAGAGAGDNLSLLLRTSSPGGAF   120
SK2               GSSPCLRCNSCASSGAP...AGAGDNLSLLLRTSSPGG.F   111
Consensus         gsspclrcnscassgap   agagdnlslllrtsspgg f

Isoform0013754    RTRTSSPLSGSSCCCCSSRRGSQLNVSELTPSSHASALRQ   160
SK2               RTRTSSPLSGSSCCCCS.RRGSQLNVSELTPSSHASALRQ   150
Consensus         rtrtssplsgssccccs rrgsqlnvseltpsshasalrq

Isoform0013754    QYAQQPASASLYHQCHSLQPAASPTGSLGSLGSGPPLSHH   200
SK2               QYAQQ.ASASLYHQCHSLQPAASPTGSLGSLGSGPPLSHH   189
Consensus         qyaqq asaslyhqchslqpaasptgslgslgsgpplshh

Isoform0013754    HHHPHLAHHQHHQSQARRESNPFTEIAMSSCRYNGGVMRP   240
SK2               H....LAHHQHHQSQARRESNPFTEIAMSSCRYNGGVMRP   225
Consensus         h    lahhqhhqsqarresnpfteiamsscrynggvmrp

Isoform0013754    LSNLSASRRNLHEMDSEAQPLQPPASVGGGGGASSPSAVA   280
SK2               LSNLSASRRNLHEMDSEAQPLQPPASVGGGG.ASSP....   260
Consensus         lsnlsasrrnlhemdseaqplqppasvgggg assp

Isoform0013754    AAASSSAPEIVVSKPEHNNSNNLALYGTGGGGSTGGGGGS   320
SK2               .SASSSAPEIVVSKPEHNNSNNLALYGTGGG......GGG   293
Consensus           asssapeivvskpehnnsnnlalygtggg      gg

Isoform0013754    GSGHGSSSGTKSSKKKNQNIGYKLGHRRALFEKRKRLSDY   360
SK2               GSGHGSSSGTKSSKKKNQNIGYKLGHRRALFEKRKRLSDY   333
Consensus         gsghgsssgtksskkknqnigyklghrralfekrkrlsdy

Isoform0013754    ALIFGMFGIVVMVIETELSWGAYDKASLYSLALKCLISLS   400
SK2               ALIFGMFGIVVMVIETELSWGAYDKASLYSLALKCLISLS   373
Consensus         alifgmfgivvmvietelswgaydkaslyslalkclisls

Isoform0013754    TIILLGLIIVYHAREIQLFMVDNGADDWRIAMTYERIFFI   440
SK2               TIILLGLIIVYHAREIQLFMVDNGADDWRIAMTYERIFFI   413
Consensus         tiillgliivyhareiqlfmvdngaddwriamtyeriffi

Isoform0013754    CLEILVCAIHPIPGNYTFTWTARLAFSYAPSTTTADVDII   480
SK2               CLEILVCAIHPIPGNYTFTWTARLAFSYAPSTTTADVDII   453
Consensus         cleilvcaihpipgnytftwtarlafsyapstttadvdii

Isoform0013754    LSIPMFLRLYLIARVMLLHSKLFTDASSRSIGALNKINFN   520
SK2               LSIPMFLRLYLIARVMLLHSKLFTDASSRSIGALNKINFN   493
Consensus         lsipmflrlyliarvmllhsklftdassrsigalnkinfn

Isoform0013754    TRFVMKTLMTICPGTVLLVFSISLWIIAAWTVRACERYHD   560
SK2               TRFVMKTLMTICPGTVLLVFSISLWIIAAWTVRACERYHD   533
Consensus         trfvmktlmticpgtvllvfsislwiiaawtvraceryhd

Isoform0013754    QQDVTSNFLGAMWLISITFLSIGYGDMVPNTYCGKGVCLL   600
SK2               QQDVTSNFLGAMWLISITFLSIGYGDMVPNTYCGKGVCLL   573
Consensus         qqdvtsnflgamwlisitflsigygdmvpntycgkgvcll

Isoform0013754    TGIMGAGCTALVVAVVARKLELTKAEKHVHNFMMDTQLTK   640
SK2               TGIMGAGCTALVVAVVARKLELTKAEKHVHNFMMDTQLTK   613
Consensus         tgimgagctalvvavvarkleltkaekhvhnfmmdtqltk

Isoform0013754    RVKNAAANVLRETWLIYKNTKLVKKIDHAKVRKHQRKFLQ   680
SK2               RVKNAAANVLRETWLIYKNTKLVKKIDHAKVRKHQRKFLQ   653
Consensus         rvknaaanvlretwliykntklvkkidhakvrkhqrkflq

Isoform0013754    AIHQLRSVKMEQRKLNDQANTLVDLAKTQNIMYDMISDLN   720
SK2               AIHQLRSVKMEQRKLNDQANTLVDLAKTQNIMYDMISDLN   693
Consensus         aihqlrsvkmeqrklndqantlvdlaktqnimydmisdln

Isoform0013754    ERSEDFEKRIVTLETKLETLIGSIHALPGLISQTIRQQQR   760
SK2               ERSEDFEKRIVTLETKLETLIGSIHALPGLISQTIRQQQR   733
Consensus         ersedfekrivtletkletligsihalpglisqtirqqqr

Isoform0013754    DFIEAQMENYDKHVAYNAERSR                     782
SK2               DFIEAQMENYDKHVAYNAERSR                     755
Consensus         dfieaqmenydkhvaynaersr
```

Figure S10. Several published genes were compared with full-length transcripts, the comparative result of the *SK2* gene.

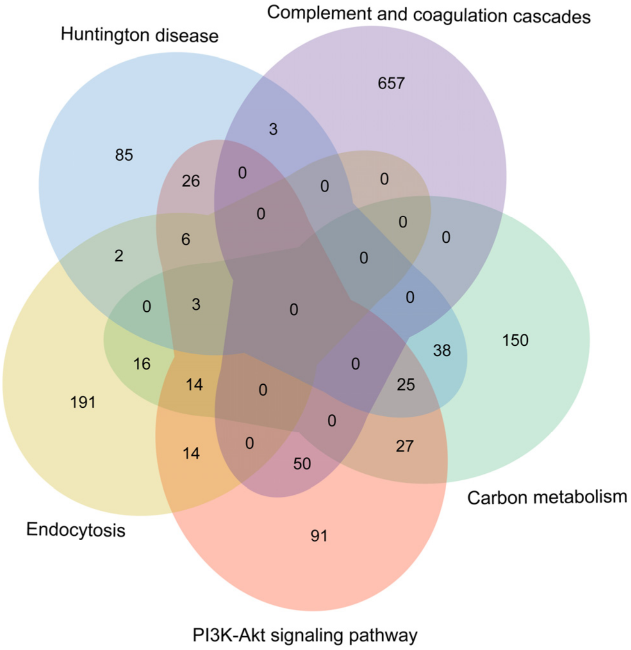

Figure S11. The number of overlapped transcripts among the top five immune pathways with the largest number of genes in KEGG annotation.
